# The Therapeutic Potential of Dietary Phytochemicals in Age-Related Neurodegenerative Disorders

**DOI:** 10.3390/ph18091268

**Published:** 2025-08-26

**Authors:** Boluwatife Olamide Dareowolabi, Eun-Yi Moon, Jin Hee Kim

**Affiliations:** Department of Integrative Bioscience & Biotechnology, Institute of Bioscience, Sejong University, Seoul 05006, Republic of Korea; doboluwatife@sju.ac.kr (B.O.D.); eunyimoon@sejong.ac.kr (E.-Y.M.)

**Keywords:** aging, neurodegeneration, phytochemicals

## Abstract

In recent times, neurodegenerative diseases have become a global health concern, particularly among the elderly. This may be attributed to the increased risk of neuronal death due to age. Moreover, the underlying mechanisms of neurodegeneration are largely driven by age-related processes that include oxidative stress, mitochondrial dysfunction, and inflammation. Despite extensive research efforts, however, neurodegenerative disorders still remain incurable as current therapeutic strategies provide limited efficacy as well as severe side effects. For these reasons, dietary phytochemicals are being considered as preventive strategies because they have potential neuroprotective functions against age-related neurodegeneration. This review summarizes the mechanisms underlying age-related neurodegeneration and highlights the current challenges in their treatment and management. It also discusses the potential of dietary phytochemicals as complementary interventions, focusing on their neuroprotective functions and mechanisms of action. Finally, challenges surrounding the use of dietary phytochemical interventions in controlling age-related neurodegenerative disorders are addressed and solutions to these challenges based on available research are discussed.

## 1. Introduction

Due to significant socioeconomic development worldwide and declining fertility rates, the global population has seen a significant shift towards an older, aging population with people aged 65 and above projected to rise from 9% to approximately 16% of the worldwide population by the year 2050 [1,2,3,4]. Population aging, however, has increased the burden of diseases globally, as age is linked to numerous diseases including neurodegenerative disorders, cancers, and cardiovascular diseases [3,5].

Neurodegenerative disorders, such as Alzheimer’s disease (AD) and Parkinson’s disease (PD) contribute to the declining health-related quality of life among aging and aged populations around the world, with more than 55 million people suffering from dementia worldwide [6,7,8]. Due to the degenerative nature of these disorders, people who suffer from neurodegenerative disorders require constant care and management, which creates a high economic burden on healthcare systems worldwide [8]. The need to effectively manage neurodegenerative disorders has led to the search for more cost-effective and readily available treatments to reduce the burden on both sufferers and the global healthcare system [9].

Phytochemicals are biologically active compounds present as complex or conjugated forms in plants [10]. However, their availability is influenced by several factors, including texture, food processing methods, and metabolism, and decades of research suggest that their antimicrobial, anti-inflammatory, and antioxidant properties may be beneficial in ameliorating age-related disorders [11,12].

An understanding of the mechanisms by which age drives the progressive decline of neuronal functions lays the groundwork for preventive and management strategies [13]. The objective of this study is to summarize existing evidence on the therapeutic potential of dietary phytochemicals in preventing and alleviating symptoms of age-related neurodegenerative disorders.

## 2. Relationship Between Aging and Neurodegenerative Disorders

Aging is the most significant and unmodifiable risk factor for all degenerative neurological diseases [14]. As cells age, cellular and subcellular changes accumulate, ultimately affecting brain physiology [15]. Furthermore, genetic mutations and epigenetic changes that impair proteostasis and exacerbate mitochondrial dysfunction are accumulated by aging cells [16]. When proteostasis is disrupted, pathogenic proteins such as amyloid beta (Aβ), α-synuclein, huntingtin, transactive response DNA-binding protein 43 (TDP-43), and hyperphosphorylated tau (p-tau) accumulate within the cell [13]. These protein aggregates contribute to the pathogenesis of neurodegenerative diseases by triggering various pathological mechanisms, including chronic neuroinflammation [13].

### 2.1. Hallmarks of Aging as Drivers of Neurodegeneration

**Figure 1:**
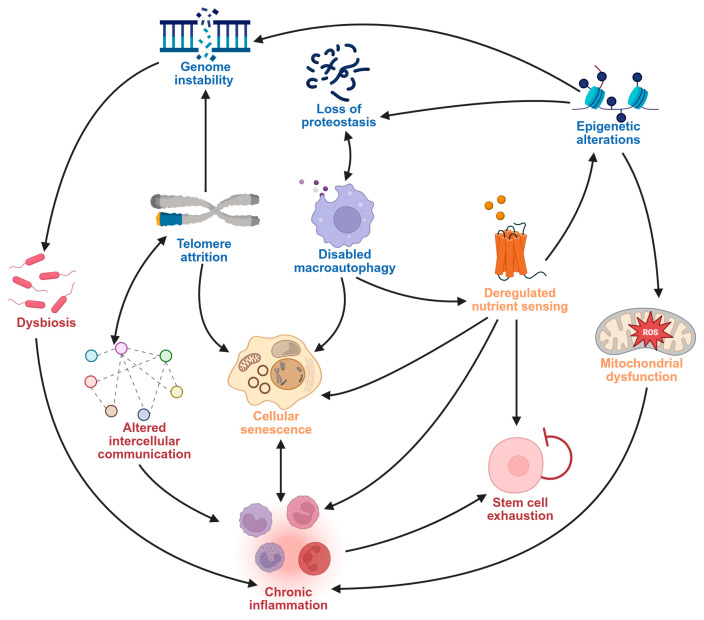
Interrelatedness of aging hallmarks.

The 12 hallmarks of aging established by Lopez Otín et al. [17] are classified into three groups: primary, antagonistic, and integrative hallmarks. These aging hallmarks interact and influence each other (Figure 1) and have been linked to the etiology and progression of age-related neurodegenerative disorders [17,18].

The primary aging hallmarks arise from accumulated damage to the genome, epigenome, and organelles [17,18,19,20,21,22,23,24,25,26] and include genomic instability, telomere attrition, disabled macroautophagy, epigenetic alterations, and loss of proteostasis [17,18]. These primary aging hallmarks promote senescence [27], disrupt longevity networks [18,28,29,30], and promote aggregation of misfolded proteins [31,32] and mitochondrial dysfunction [33], all of which characterize age-linked neurodegeneration.

Antagonistic aging hallmarks arise in response to the damage incurred from the primary hallmarks of aging [17,18] and they include cellular senescence, mitochondrial dysfunction, and deregulated nutrient sensing. Senescent cells resist apoptosis and accumulate dysfunctional mitochondria, retaining an active, but dysfunctional metabolic profile [34,35] that promotes increased reactive oxygen species (ROS) production [36]. In addition, aging also compromises the somatotrophic axis, disrupting intracellular nutrient sensing and signaling [37].

Integrative aging hallmarks which arise due to damage accumulated from both primary and antagonistic hallmarks of aging control the rate at which aging-related changes occur [17,18]. Human microbiome dysbiosis, stem cell exhaustion, altered intercellular communication, and chronic inflammation are the integrative aging hallmarks [17,18]. Aging alters the microbiome balance of the human host, reducing individual and larger inter-individual microbial diversities and, thereby, contributing to dysbiosis [38]. Aging impairs the ability of tissues to regenerate by weakening stem cells [39]. Aging also disrupts intercellular communication and cells’ immunosurveillance activities, leading to increased inflammation and inflammaging, a major factor in the development and progression of neurodegenerative diseases [31].

### 2.2. Age-Related Mechanisms in Neurodegenerative Disorders

#### 2.2.1. Major Neurocognitive Disorder

Major neurocognitive disorder, which is more commonly known as dementia, is a common neurodegenerative disorder impacting mobility and responsiveness [40]. It has a collection of symptoms that impact cognitive ability, memory, and mobility [41]. Aging is a vital predisposing factor for dementia and, although it can present as early-onset disease in people under the age of 65, most dementia disease cases occur in people aged 65 and older [42]. Deregulated proteostasis, inflammation, and dysfunctional metabolism are some of the mechanisms involved in the development of dementia [43,44,45]. Depending on the underlying pathology and symptoms, as well as affected brain regions, there are different types of dementia [42].

AD, the most prevalent form of dementia among older people, exhibits loss of cholinergic neurons and progressive impairments in cognition, behavior, and motor abilities [44]. AD is primarily caused by increased accumulation of tau-protein and Aβ, peptides that form tangles and plaques in the diseased brain [20]. In addition, as aging progresses, ROS increase and contribute to increased lipid peroxidation, protein oxidation, and mitochondrial dysfunction [20]. These cellular events may induce synaptic failure and consequently lead to the development of AD [44]. Reduced energy metabolism due to impaired nutrient sensing is also a distinctive feature of AD [44].

The second most common form of dementia is dementia with Lewy bodies (DLB) [45]. This is known to predominantly affect individuals over the age of 50 [45]. It is induced by an accumulation of Lewy bodies, which are primarily composed of α-synuclein proteins that alter brain chemistry and encourage neuroinflammation [45]. Hallucinations, sleep disturbances, muscle tightness, and cognitive loss are all linked to DLB [45].

Vascular dementia (VaD) is another age-related form of dementia affecting individuals over the age of 65 [43]. Cognitive impairment caused by ischemic or hemorrhaging brain injury is a hallmark of VaD [43]. Increasing age is a risk factor of VaD [43]. Several pathological mechanisms that are elevated in aging cells, such as oxidative stress, apoptosis, inflammation, and autophagy, are also increased in patients with VaD [43].

#### 2.2.2. Parkinsonism-Type Diseases (PTD)

PTD is a range of movement disorders, including PD, caused by a reduction in cells and nerve fibers that produce dopamine and norepinephrine [46]. They are characterized by different motor symptoms, including bradykinesia (slow movement), stiffness of muscles, rest tremors, and difficulties in maintaining posture and gait [46]. Other non-motor symptoms are cognitive decline, olfactory dysfunction, pain, and sleep disorders [46]. Molecular pathology of PTD involves accumulation of α-synuclein in the brain due to age-related decline in the brain’s proteolytic capacity [46]. Mitochondrial dysfunction, which increases with age, leads to the degeneration of dopaminergic fibers, increases the production of ROS, and also serves as a causal mechanism in PTD [46]. The brains of those who suffered from PTD show high levels of indicators for neuroinflammatory damage in post-mortem studies [47]. As the mechanism by which neuroinflammation may cause PTD is not yet fully understood, researchers have hypothesized that α-synuclein aggregation may cause a shift towards pro-inflammation, which contributes to neuronal degeneration, further promoting the depletion of dopamine [47]. Another mechanism contributing to PTD is gut dysbiosis [48]. Inflammatory mechanisms in the enteric nervous system may be mediated by the gut microbiome, contributing to chronic systemic inflammation that further promotes the development of Parkinsonism [48].

#### 2.2.3. Motor Neuron Diseases (MNDs)

MNDs comprise a group of disorders defined by muscle weakness and atrophy as a result of damage to the motor neurons [49]. These diseases include amyotrophic lateral sclerosis (ALS), primary lateral sclerosis, spinal muscular atrophy, progressive bulbar palsy, and progressive muscular atrophy [49]. ALS is the most prevalent motor neuron disease and aging is one of its most notable risk factors [50]. In addition to chronological aging, several age-related factors, like increased oxidative stress, impaired protein homeostasis, and inflammaging, are contributors to the development and progression of ALS [50]. With progression in age, the mitochondria in motor neurons become increasingly dysfunctional [50]. The dysfunction in mitochondria, which leads to reduced mitochondrial DNA copy number and cell body size, affect the function and survival of motor neurons, contributing to ALS pathology [50]. Research has shown that aging affects various motor unit cells, which include motor neurons, skeletal muscle cells, astrocytes, and Schwann cells [50]. Aging in these cells promotes neuroinflammation, senescence, decline in motor unit function, and aberrance in mitochondria, all of which promote ALS [50].

#### 2.2.4. Huntington’s Disease (HTTD)

HTTD is an autosomal dominant neurodegenerative disorder resulting from a mutation in the *Huntingtin* (*HTT*) gene [51]. The mutated *HTT* gene, which is translated into a mutated huntingtin protein, disrupts normal cellular processes and results in the dysfunction of the basal ganglia striatum and the cerebral cortex [51]. Although every case of HTTD is a consequence of the mutated *HTT* gene, its clinical onset and progression are related with biological aging [51]. Evidence from research has shown that leukocytes in those suffering from HTTD have shortened telomeres [51]. In HTTD, inflammation increases as a result of altered intercellular communication [51]. Additionally, people suffering from HTTD show signs of hypermetabolism which may be due to dysregulated nutrient sensing [51].

## 3. Current Treatment and Management Strategies of Age-Related Neurodegenerative Disorders

A defining feature of neurodegenerative disorders is progressive neuronal loss [52]. Although recent research has demonstrated that limited neurogenesis occurs in some regions of the normal adult brain, most nerve cells are irreplaceable [52]. As a result, neurodegenerative disorders are currently incurable [9]. Treatment of age-related neurodegenerative disorders involves management of symptoms through pharmacological and non-pharmacological interventions in order to alleviate disease progression and prolong life [9]. Pharmacological interventions involve the use of approved medications to manage symptoms and potentially slow down neurodegeneration [9]. Non-pharmacological interventions, on the other hand, include lifestyle modifications, physical therapies, and cognitive training to slow down functional decline, improve cognition, and enhance quality of life [9].

### 3.1. Pharmacological Interventions in the Management of Age-Related Neurodegenerative Disorders

Conventional treatment strategies for neurodegenerative disorders include pharmacological interventions like cholinesterase inhibitors (ChEIs), N-methyl-D-aspartate (NMDA) receptor antagonists, dopaminergic agents, and antipsychotic drugs [53].

Donepezil, rivastigmine, and galantamine are the most common ChEI-based medications approved for neurodegenerative disorders like dementia and PTD [54]. Cholinergic neurons play necessary roles in learning, memory, and other cognitive functions [54]. With aging, cholinergic neurons decrease, reducing brain cholinergic transmission and thereby increasing the risk of dementia and other neurodegenerative diseases [54]. ChEIs prevent the breakdown of acetylcholine, the neurotransmitter released by cholinergic neurons, to improve cognitive and motor functions [54].

NMDA receptors are glutamate receptors that function in central nervous system (CNS) development, neuroplasticity, learning, and memory [55]. Because overstimulation of NMDA receptors has been implicated in neurodegeneration, they may also be targeted in the management of age-mediated neuronal disorders [55]. Memantine, the most commonly prescribed NMDA receptor antagonist, is often used alongside ChEIs in the management of AD [55].

Dopaminergic agents like levodopa, pramipexole, and ropinirole are often prescribed to increase dopamine levels and thus improve cognitive and motor functions in PD [56].

Antipsychotic drugs are widely used to alleviate symptoms of psychotic disorders like schizophrenia, mania, and depression [57]. Antipsychotic drugs are classified as either typical or atypical [57]. Typical antipsychotics are D2 dopamine receptor antagonists which inhibit dopamine neurotransmission [58]. Because treatment with typical antipsychotics may elevate the risk of neurological adverse effects like dystonia or akathisia, atypical antipsychotics are a preferred treatment option [58]. Atypical antipsychotic drugs like aripiprazole, olanzapine, and risperidone are often prescribed to reduce the behavioral and psychological symptoms that accompany neurodegenerative disorders, as they have lesser risks of neurological side effects [59].

Medications for neurodegenerative disorders are mostly symptom improving and do not cure or reverse neurodegeneration [60]. Additionally, the effects of these medications are often reversible and become less effective as disease progresses [60]. As a result of these limitations, research on pharmacological interventions for neurodegeneration is constantly evolving with new treatments [61]. In 2023, lecanemab was approved for the treatment of mild cognitive impairment and AD [61]. Lecanemab targets Aβ protofibrils, pre-fibrillar forms of Aβ that are involved in the development of AD [62]. These protofibrils alter membrane integrity by inducing oxidative stress, causing dysregulation of intracellular calcium, and causing synaptic toxicity [62]. By binding Aβ, lecanemab decreases pathogenic Aβ and its deposition [63]. Donanemab is a monoclonal antibody recently approved for management of early-onset AD in adults [64]. Clinical trials conducted with this medication have reported that treatment with donanemab results in slower cognitive and functional decline in people with early onset symptomatic AD [64]. Donanemab targets N3pG, a modified form of Aβ found in the amyloid plaques of those suffering from AD [65]. The interaction of donanemab with N3pG activates microglia, which promotes clearance of plaques from the neurons, thus decreasing amyloid load and toxicity [65]. To improve motor fluctuations in adults with PD, the U.S. Food and Drug Administration (FDA) has approved a new therapy known as Onapgo [66]. Onapgo is a wearable device that constantly delivers apomorphine as an under-the-skin infusion throughout the day [66]. Apomorphine is a non-selective dopamine agonist that activates all dopamine receptor sub-types, increasing dopaminergic activity and alleviating motor symptoms in PD [67]. Tofersen (Qalsody), an antisense oligonucleotide, was also approved in 2023 for the treatment of ALS [68]. It is the first FDA-approved medication targeting a genetic cause of ALS [68]. About 2% of ALS cases in adults are due to a genetic mutation in the *superoxide dismutase 1* (*SOD1*) gene [68]. Tofersen binds to mutated *SOD1* mRNA, suppressing the translation of dysfunctional SOD1 protein [68].

Although these newly approved medications have represented important advances in the treatment of neurodegeneration, information on their long-term benefits and safety still remain limited, thus extensive studies are required to fully explore their clinical impacts [69].

While current pharmacological interventions provide temporary symptomatic relief, their side effects and limited efficacy reinforce the urgent need for more permanent solutions to age-linked neurodegeneration, as shown in Table 1. In addition, while the more recent FDA approval of advanced therapies like donanemab, lecanemab, and tofersen represent encouraging steps towards more targeted, mechanism-based therapies, several research gaps still persist. Efficacy, long-term side effects, drug interaction, and cost efficiency of new medications will ultimately determine their clinical usability and mainstream adoption.

### 3.2. Non-Pharmacological Interventions in the Management of Age-Related Neurodegenerative Disorders

Non-pharmacologic interventions in alleviating symptoms of neurodegeneration typically involve lifestyle modifications to enhance patients’ quality of life [70].

One of the most common interventions for the alleviation of neurodegenerative symptoms is physical activity [71]. Research has shown that exercise helps decrease circulating pro-inflammatory factors, thereby reducing inflammation, a major mechanism for the progression of neurodegeneration [71]. Exercise also promotes IGF-1 functions, which contribute to better nutrient sensing and improved metabolic profile in diseased brains [72].

Cognitive training alongside mental stimulation techniques like electroconvulsive therapy and deep brain stimulation are sometimes provided for people suffering from neurodegeneration to improve their cognitive abilities [70].

Dietary interventions like the Mediterranean or ketogenic diets have yielded favorable outcomes in ameliorating oxidative stress and inflammation, improving gut microbiome—and thus reducing neuron atrophy—and improving cognitive abilities in individuals diagnosed with dementia and PD [70]. Some studies have also suggested that caloric restriction and intermittent fasting may help improve the brain’s metabolic profile, reduce neurodegeneration, and improve overall brain health [73].

New interventions to improve symptoms of neurodegeneration include gene therapy and stem cell therapy [74,75]. Research on gene therapy for neurodegenerative disorders involves the replacement of genes that predispose people to neurodegenerative disorders or the addition of genes that improve resistance to neurodegeneration [74]. Clinical trials in PD are aimed at improving dopamine levels through adeno-associated virus-mediated gene therapy [74]. This approach involves either the transfer of glutamic acid decarboxylase for the synthesis of γ-aminobutyric acid from glutamate or the gene transfer of aromatic L-amino acid decarboxylase, an enzyme for dopamine synthesis [74]. Because cholinergic neuron activities are improved by nerve growth factor (NGF), research on the delivery of the gene encoding NGF has been studied as a therapeutic option for improving cognitive function in people suffering from AD [74]. Although preclinical studies in AD animal models showed that exogenous NGF delivered into the brain via an adeno-associated virus vector (AAV2–NGF) improved cholinergic activities, phase 1 and 2 trials in humans have not produced any improvements in cognition [74]. Brain autopsy results from the human study revealed that AAV2–NGF did not reach the target cholinergic neurons [74]. Therefore, the efficacy of this approach has not been definitively proven in humans [74].

Regenerative stem cell therapy is another option being considered as an alternative to conventional treatment methods of neurodegenerative disorders [75]. Stem cells have the ability to develop into different cell types, which makes them potentially instrumental in repairing injured neuronal cells, enhancing cognition and protecting healthy neurons from degeneration [75]. Although research in stem cell therapy for neurodegeneration is still in its early stages, research in animal models has produced successful results [75]. In a study utilizing animal models of PD, genetically engineered mesenchymal stem cells encoding three genes for the synthesis of dopamine were transplanted into rats [76]. These stem cells restored dopamine levels, thus reconstructing dopamine pathways in the mid-brain [76]. In another study, induced pluripotent stem cells (iPSCs) obtained from mouse skin fibroblasts were transplanted into a mouse model of AD [77]. Results from the study showed that the iPSCs differentiated into glial cells and also caused a decrease in the deposition of Aβ plaques [77].

Lifestyle modifications offer a low-risk and low-cost option for the elderly; however, this option is more preventive than curative [78]. Moreover, these modifications, even as a preventative measure, depend heavily on the consistency of the patients and are unlikely to alter the progression of genetically driven conditions like Huntington’s disease [79]. In addition, while other non-pharmacological options, including gene therapy and regenerative stem cell therapy, have shown promise, the evidence for their efficacy and safety in humans remains limited [74,75]. As a result, pharmacological measures remain the most acceptable therapeutic option for alleviating symptoms of neurodegeneration in the elderly.

### 3.3. Limitations in Current Intervention Methods for the Management of Age-Related Neurodegenerative Disorders

Despite the efforts being expended by researchers and medical professionals in the development of both pharmacological and non-pharmacological interventions, neurodegenerative disorders are still among the top causes of mortality and morbidity among the elderly [80]. Current management techniques have several limitations that reduce their efficacy and effectiveness [9].

First, most drugs approved for the management of neurodegeneration have many adverse effects, which further contribute to the lowering of the health-related quality of life among the aging population [9]. Levodopa, the first dopaminergic agent used in the treatment of PD, increased the risks of dyskinesias (involuntary muscle movements), nausea, and confusion [81]. Olanzapine, an antipsychotic drug, has also been reported to demonstrate severe side effects on the gut, inducing weight gain, disturbing the metabolic profile, and ultimately leading to gut dysbiosis [82].

Second, because of the semi-permeable nature of the blood–brain barrier (BBB), a cellular barrier that regulates the exchange of substances between the brain and bloodstream, the delivery of therapeutic compounds to the brain remains a challenge [83]. The BBB may prevent the movement of medicines to their targets, thus preventing the pharmacological agents from successfully preventing the progression of neurodegeneration [83].

Third, apart from pharmacological agents, non-medicinal methods also have significant limitations. Dietary-related interventions, including restrictions, could increase the risks of unwanted side effects like drastic weight loss and nutrient deficiencies [84].

Fourth, while new interventions are constantly being researched, approval by appropriate regulatory bodies is needed before they can be made available for use as treatment options for neurodegenerative disorders [9]. In addition, these newer interventions might not be cost-effective for most elderly people if and when they are approved [9].

Due to the different side effects and limitations for current treatment options for neurodegeneration, there is a need for other novel, readily available and cost-effective treatment options. One option currently under research is that of dietary phytochemicals.

## 4. Dietary Phytochemicals in Age-Related Neurodegenerative Diseases

### 4.1. Types, Properties, and Anti-Aging Activities of Dietary Phytochemicals

Phytochemicals are secondary metabolites of plants and serve crucial functions in the plant lifecycle [85]. Phytochemicals may be involved in protection and reproductive functions, as well as the production of hormones necessary for growth, signaling, and defense [10]. When consumed by humans, phytochemicals have been proven to promote physiological functions like antioxidant or anti-inflammatory activities [10].

Dietary phytochemicals are bioactive plant chemicals that are naturally present in plant-based food [86]. Several dietary phytochemicals have been isolated and studied for their efficacy in the management of chronic and non-communicable diseases [87]. Despite the numerous dietary phytochemicals available, they can be grouped into polyphenols, carotenoids, glucosinolates, terpenoids, organosulfur compounds, and nitrogenous compounds [87].

Polyphenols are bioactive products produced by plants as a defense mechanism against damage from radiation or microbial infection [88]. Polyphenols show structural variability and are classified as flavonoids (which are further classified as flavanols, isoflavones, anthocyanins, flavanones, flavones, and flavonols), phenolic acids, stilbenes, and tannins based on the number of phenol rings and hydroxyl groups in their structure [89]. Research has shown that polyphenols, which are available in vegetables, citrus fruits, berries like cranberries, blueberries, and blackberries, may confer anti-inflammatory and anti-bacterial protection to neurons, thereby reducing the risks of both neuroinflammation and microbial dysbiosis [88].

Carotenoids are natural pigments found in plants responsible for the bright colors of fruits and vegetables [90]. Carotenoids have been studied for their health benefits, especially in ocular health [90]. These pigments have high antioxidant properties and thus have shown promise in the improvement of cognitive functions [90]. Carotenoids can be further classified as either carotenes or xanthophylls [91]. The most common carotenoids in human diets are sourced from carrots, tomatoes, papayas, and corn [90].

Glucosinolates are dietary phytochemicals found predominantly in cruciferous vegetables like cauliflower, broccoli, arugula, and bok choy that contain sulfur and nitrogen [92]. Upon ingestion of plants containing glucosinolates, enzymatic breakdown occurs, metabolizing this bioactive compound into metabolites like sulforaphane and isothiocyanates [92]. These metabolites may provide protection to neurons via their antioxidative and anti-inflammatory functions [92].

Terpenoids are regarded as the most abundant and diverse groups of natural products [93]. They are secondary plant metabolites with a wide range of pharmacological functions, including anti-microbial, anti-inflammatory, and hypoglycemic activities [93]. These characteristics of terpenoids support the idea that they may provide protection against age-related diseases like neurodegeneration [93]. The sources of terpenoids include *Paeonia lactiflora*, inula flower, and ginseng [93]. These plants are staples in Asian traditional medicine, particularly ginseng [93].

Organosulfur compounds are organic compounds containing sulfur [94]. These bioactive compounds have been studied for their anticarcinogenic properties [94]. They are also considered to regulate pathways important for cellular regulation due to their antioxidative and anti-inflammatory functions [94]. Organosulfur compounds like allicin are found in garlic bulbs [94].

Nitrogenous phytochemicals are a diverse group of secondary plant metabolites with one or more nitrogen atoms in their structural formula [12]. These bioactive compounds may prevent neuronal damage due to their antioxidative and anti-inflammatory properties [12]. Some sources of nitrogenous phytochemicals include coffee, tobacco, and tea [87].

Dietary phytochemicals may regulate antiapoptotic proteins and activate intracellular pathways, while suppressing oxidative enzymes and enhancing mitochondrial functions, thus reducing neurological inflammation and oxidative stress, and consequently improving cognition [95].

Aging is linked to a progressive loss of function in organ systems, which contributes to morbidity and mortality [96]. Recently, aging research has focused on the development of anti-aging strategies that include pharmacological agents, lifestyle interventions, and the use of dietary phytochemicals [97].

Dietary phytochemicals possess multiple health-related functions and have been studied extensively for their potential anti-aging properties [98]. Due to their antioxidant and anti-inflammatory properties, dietary phytochemicals may modulate cellular processes underlying aging, thereby mitigating the progression of age-related diseases [98]. Research on dietary phytochemicals has shown that polyphenols, like quercetin, genistein, ferulic acid, and apigenin, possess anti-inflammatory and antioxidant functions which may slow down aging and alleviate the symptoms of age-related diseases [99,100,101,102]. In animal models, fisetin, a flavonol found in fruits and vegetables, has been shown to suppress the upregulation of some aging markers [103]. Resveratrol may regulate apoptosis while protecting the cells against oxidative stress and inflammation [104]. Other dietary phytochemicals, like tomatidine, glucoraphanin, and lycopene, also possess anti-aging properties, as they prevent mitochondrial damage and protect the cells from ROS [105,106,107]. Additionally, dietary phytochemicals like hesperidin and pinoresinol improve immune functions and protect DNA from damage [108,109]. Table 2 summarizes the anti-aging effects of dietary phytochemicals in preclinical studies [99,100,101,102,103,104,105,106,107,108,109].

### 4.2. Bioavailability and Transport Mechanism of Dietary Phytochemicals for Neuroprotection

#### 4.2.1. Bioavailability of Dietary Phytochemicals

Dietary phytochemicals are predominantly consumed via food and are present in complex or conjugated forms within the vacuoles of plant cells [86]. Their potential neuroprotective functions are largely influenced by digestion, metabolism, and their ability to reach the neurons [110]. Although some dietary phytochemicals like quercetin possess beneficial qualities in their native forms, others must undergo extensive metabolism into metabolites to elicit biological advantages [111].

Metabolism of dietary phytochemicals involves digestion, absorption, enzymatic transformation by enterocytes and liver enzymes, and modulation by the gut microbiome [112]. These metabolic processes impact the bioavailability and biological functions of phytochemicals [112]. Typically, parent phytochemicals reach systemic circulation in low concentrations due to poor absorption, whereas their metabolites show higher concentration levels in circulation [112]. These metabolites of dietary phytochemicals show better pharmacokinetic profiles, including improved absorption and greater permeability across the BBB when compared with their parent compounds [112,113]. Therefore, effective phytochemical treatment strategies should consider both direct effects of native phytochemicals and the biological effects of their metabolites in providing neuroprotective functions in neurodegenerative disorders.

#### 4.2.2. Transport of Dietary Phytochemicals to the Brain

Once in circulation, dietary phytochemicals and their metabolites may impact neuronal signaling pathways, either directly by crossing the BBB or indirectly via mechanisms like the gut–brain axis (GBA) [111].

##### Transport of Dietary Phytochemicals Across the BBB

The BBB is a multicellular layer that acts as a barrier between the peripheral circulation and the CNS [83]. Although the BBB is essential for maintaining CNS integrity by limiting the entry of toxins into the brain, it may also prevent the passage of beneficial compounds like dietary phytochemicals into the CNS [114]. Physicochemical properties like lipophilicity, molecular weight, molecular flexibility, topological surface area, and hydrogen ion binding capacity influence how dietary phytochemicals penetrate the BBB [114]. In normal physiological conditions, substances can be transported across the BBB via passive diffusion, receptor-mediated transport, adsorptive transcytosis, and carrier-mediated transport [115]. Some highly lipophilic phytochemicals with smaller molecular weight (<400 Da) permeate the BBB via passive diffusion [116,117]. For instance, quercetin (302.24 Da), a flavonol found in fruits and vegetables, has the capacity to permeate the BBB via passive diffusion [115]. This dietary phytochemical provides neuroprotection via its ability to scavenge free radicals and prevent oxidative stress [118]. Paeonol, a dietary phytochemical sourced from the root bark of Cortex Moutan, crosses the BBB via a carrier-mediated transporter system despite its low molecular weight (166.17 Da) and high lipophilicity [119]. Other phytochemicals may bind to surface receptors or utilize adsorptive transcytosis to cross the BBB [83], and though data on these routes still remain sparse, they highlight important areas for future research.

##### Contribution of the GBA in Dietary Phytochemical-Based Neuroprotection

Dietary phytochemicals may also provide neuroprotection via the GBA [115]. The GBA is a two-way communication system that connects the brain to the gastrointestinal tract via hormonal, neural, and immune systems [120]. The GBA contributes to neuronal homeostasis and overall brain health [110]. The microbiota that reside in the gut influence brain activities, and vice versa [120]. Therefore, a disruption in the balance of the microbiome constituent of the gut, known as dysbiosis (a hallmark of aging), has been implicated in the onset of neurodegenerative disorders through metabolic and inflammatory mechanisms [120]. Dietary phytochemicals may exert their neuroprotective functions via the gut [121]. Dietary phytochemicals like curcumin, with proven neuroprotective functions, are limited in their efficacy due to chemical instability, poor availability, and rapid metabolism [121]. Enzymes produced by the gut microbiota have been reported to modify polyphenols to improve their absorption, thus increasing their bioavailability and efficacy [121]. Therefore, modification of curcumin into bioactive metabolites by gut microbiota enzymes may improve its efficacy and actions on the brain [122]. Additionally, bacteria strains like bifidobacteria and lactobacilli may act on dietary phytochemicals via metabolic processes that include demethylation, hydroxylation, reduction, and demethoxylation [112]. These metabolic processes produce metabolites that are absorbed more easily and may cross the BBB easier than parent phytochemicals [123].

### 4.3. Dietary Phytochemical-Based Neuroprotection

#### 4.3.1. Neuroprotective Functions of Dietary Phytochemicals: Evidence from Pre-Clinical Studies

Dietary phytochemicals have been extensively studied as a therapeutic option in the treatment of age-related neurodegenerative diseases [95]. Animal and cell line studies have shown that dietary phytochemicals may target different mechanisms and hallmarks of aging to reduce the progression of neurodegeneration and enhance the overall quality of life among the elderly [124]. Baicalein, a flavone found in the roots of the Scutellaria baicalensis plant, promotes healthy intestinal microbiome balance, while Berberine improves proteostasis and macroautophagy in animal models of AD [125,126]. Research has also shown that resveratrol and quercetin improve mitochondrial function to slow down the progression of PD, MND, and HTTD [127,128,129]. Treatment of animal models of neurodegeneration with curcumin has also shown improved neurogenesis, genomic stability, and overall improved cognition [130,131]. Table 3 shows the effectiveness of dietary phytochemicals in alleviating neurodegeneration by targeting aging hallmarks in preclinical studies [125,126,127,128,129,130,131].

#### 4.3.2. Neuroprotective Functions of Dietary Phytochemicals: Evidence from Clinical Trials

Phytochemicals have been formulated in different forms like tablets and in powdery form to be administered as medication in the management of diseases [132]. Clinical trials have been performed to investigate the penetrance and effectiveness of formulated dietary phytochemicals in the management of neurodegenerative disorders [133]. These trials have produced some favorable results, such as improved nutrient sensing and endothelial function, reduced senescence, and reduced inflammatory biomarkers [132,133,134,135]. Table 4 summarizes the clinical trials conducted in humans exploring the therapeutic potential of dietary phytochemicals in managing symptoms of neurodegenerative disorders [132,133,134,135,136,137,138].

Although most of the clinical studies shown in Table 4 report promising effects of dietary phytochemicals in alleviating neurodegenerative symptoms, these findings should be interpreted with caution. This is because of a major limitation in most of the trials: small sample size. Although two of the studies enrolled over a hundred participants, the remaining five clinical trials had less than fifty participants, such as that of Millar et al. [134], which included only twelve participants. Such small cohorts hinder the ability to draw conclusive evidence regarding the neuroprotective efficacy of phytochemicals in humans. At best, these studies provide a preliminary framework that warrants validation through larger and more intensive clinical studies.

#### 4.3.3. Mechanistic Pathways of Dietary Phytochemical-Based Neuroprotection

Phytochemicals have been extensively studied for several age-related diseases, particularly cancer and neurodegeneration [95]. These compounds work by influencing pathways related to inflammation, cellular stress, and mitochondrial function [139]. Some of these pathways include the oxidative stress response-related nuclear factor E2-related factor 2 (Nrf2) pathway, nuclear factor κ-light-chain-enhancer of activated B cells (NF-κB) pathway, and Parkin/phosphatase and tensin homolog (PTEN)-induced putative kinase 1 (PINK1) pathway [139,140,141].

The transcription factor Nrf2 plays a major role in regulating cellular defense mechanisms against oxidative stress and neuroinflammation [141,142]. In normal conditions, Nrf2 is bound to Kelch-like enoyl-CoA hydratase-associated protein 1 (Keap1), a repressor protein in a transcriptionally inactive state in the cytoplasm [141,142]. In the CNS, Nrf2 is widely expressed and coordinates the transcription of neuroprotective proteins [142]. As aging progresses, however, pro-inflammatory factors and ROS accumulate in the cell, causing Nrf2 to be separated from its repressor protein and translocated to the nucleus [143]. In the nucleus, Nrf2, together with small Maf protein, binds to antioxidant response elements or electrophile response elements to mediate the defense against oxidative stress [143]. In neurodegenerative disorders, Nrf2 translocation to the nucleus is reduced [144]. Phytochemicals may help activate the Nrf2 pathway to reduce oxidative stress and promote cognitive function [145]. Phytochemicals like resveratrol increase the nuclear translocation of Nrf2 by inhibiting Keap1, increasing antioxidant activity and protecting against age-related neurodegenerative diseases [146]. In a study in which cytotoxicity was induced in PC12 cells by amyloid-β_1-42_, treatment with resveratrol, a phytochemical found in grapes, attenuated cell loss and reduced oxidative stress [147]. The results of this study showed that resveratrol promoted the nuclear translocation of Nrf2, resulting in the upregulation of the antioxidant gene *HO-1* [147,148].

NF-κB transcription factors regulate cellular signaling pathways that mediate inflammatory responses [149]. The classical and alternative NF-κB pathways control cellular responses to inflammation through the breakdown of inhibitor of NF-κB (IκB) proteins [149]. Phytochemicals have been shown to modulate these NF-κB pathways by inhibiting the ubiquitination and phosphorylation of upstream signaling molecules, as well as preventing the degradation of IκB proteins [150]. This blocks NF-κB from translocating into the nucleus and causes a reduction in the transcription of pro-inflammatory genes [150]. For instance, curcumin and resveratrol suppress the phosphorylation of IκBα, which prevents the nuclear translocation of the p65 NF-κB subunit [151]. Additionally, a study using the curcumin analog EF31 [3,5-Bis(2-pyridinylmethylidene)-4-piperidone] demonstrated that curcumin inhibits IκB kinase β (IKKβ), further attenuating inflammatory signaling [152]. Treatment of BV2 microglial cell lines with curcumin, one of the major phytochemicals in turmeric, reduced pro-inflammatory markers and suppressed the activation of the NF-κB pathway, promoting an anti-inflammatory phenotype [153]. Ginsenoside, a terpenoid, and the major active pharmaceutical ingredient in ginseng, inhibits NF-κB, providing anti-inflammatory effects and considerable neuroprotection [93].

**Figure 2:**
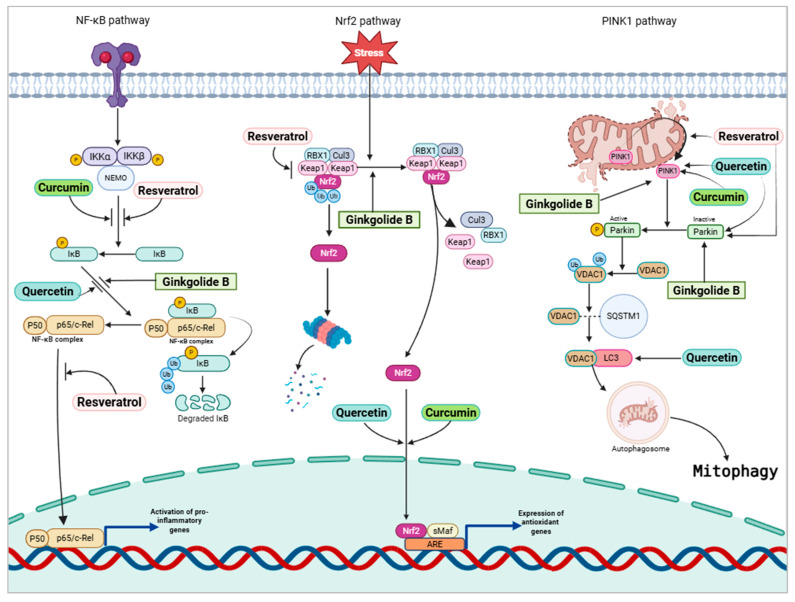
Therapeutic targets of dietary phytochemicals in pathways involved in age-related neurodegenerative disorders. c-Rel, cellular reticuloendotheliosis virus oncogene; Cul3, Cullin-3; IKK, IκB kinase; Keap1, Kelch-like enoyl-CoA hydratase-associated protein 1; NEMO, NF-κB essential modulator; NF-κB, nuclear factor kappa-light-chain-enhancer of activated B cells; Nrf2, nuclear factor erythroid 2–related factor 2; PINK1, PTEN-induced kinase 1; RBX1, RING-box protein 1; sMaf, small musculoaponeurotic fibrosarcoma oncogene homolog; VDAC1, voltage-dependent anion-selective channel 1.

Pathways involved in mitochondrial function are also targeted by dietary phytochemicals [154]. Clearing out damaged mitochondria is undertaken by a mechanism known as mitophagy [32]. Normal mitophagy is mediated by the PTEN-induced putative kinase 1 (PINK1) pathway [154]. PINK1, which is found in the inner mitochondrial membrane, is a sensor of mitochondrial damage [155]. Once mitochondrial damage is detected, PINK1 translocates to the outer mitochondrial membrane, where it causes the activation of E3 ubiquitin protein ligase (Parkin) in the cytoplasm [141]. This causes the phosphorylation of voltage-dependent anion channel 1 (VDAC1), causing it to interact with a signaling adaptor called p62/sequestosome-1 [141]. This interaction causes VDAC1 to initiate mitophagy by binding with members of the Atg8 family of homologous proteins [141]. In neurodegenerative disorders, mitophagy is impaired [141]. Aggregates of Aβ peptides and tau proteins are found in the brains of patients suffering from AD and may induce the impairment of mitophagy [155]. Misfolded tau proteins increase the membrane potential of the mitochondria, damaging the PINK1/Parkin pathway, thereby preventing normal mitophagy [155]. The anti-inflammatory and antioxidant activities of phytochemicals promote normal mitophagy [141]. Ginkgolide B, a phytochemical extracted from gingko biloba plant, has also shown promise in improving mitophagy by targeting the PINK1 pathway and alleviating neuroinflammation [156]. Treatment of Huh7 and Hep3B cell lines with quercetin, a phytochemical abundantly present in fruits and vegetables, shows enhanced expression of PINK1 and PARK2 which are key regulators of mitophagy [156,157]. Phytochemicals protect neurons against Aβ-mediated cell death and may also increase the expression of kinases and vesicles that promote normal clearing of damaged mitochondria [99]. Figure 2 shows the pathways involved in age-related neurodegeneration as well as the therapeutic targets of phytochemicals.

#### 4.3.4. Comparing the Therapeutic Potential of Dietary Phytochemicals

In this review, several dietary phytochemicals were considered with evidence from preclinical and clinical studies. In general, dietary phytochemicals provide invaluable properties like antioxidant and anti-inflammatory activities that promote neuroprotection when consumed in food [93]. However, research evidence has shown that the therapeutic efficacy and safety of these compounds vary and depend on different factors [108].

Some phytochemicals show increased efficacy when used in conjunction with other compounds [56,99]. Quercetin, for example, showed significant effects in alleviating PD symptoms in 6-hydroxydopamine (6-OHDA)-induced rats [101]. However, when combined with piperine, a compound that has been reported to improve quercetin bioavailability, the anti-PD effects of quercetin are significantly increased [101]. Other dietary phytochemicals show a dose–response relationship, with higher doses and longer durations providing greater neuroprotection [130,135]. MP showed higher mean ‘on’ time in PD patients especially in higher doses or when combined with a dopamine decarboxylase inhibitor [132].

Selecting the most promising dietary phytochemicals for the management of neurodegeneration is complicated. The studies included in this review were conducted in diverse experimental conditions, making direct comparisons limited. Nevertheless, based on the evidence synthesized in this review, we consider resveratrol, curcumin, and quercetin to be the most promising dietary phytochemicals for the management of age-linked neurodegenerative disorders. These three dietary phytochemicals have been extensively studied and have shown promising neuroprotective function across animal and human studies [101,104,127,128,129,130,131]. In contrast, phytochemicals like pinoresinol, show a weaker ability to improve oxidative function and improve neurodegeneration [109].

Overall, these studies suggest that, while dietary phytochemicals hold the promise of a safe and effective management option, they are better suited as adjunctive therapies rather than standalone treatments [132]. This is because their effectiveness seems to depend heavily on dosage, interaction with other active pharmaceutical ingredients, and treatment duration [99,130,135,136]. While their dietary phytochemicals are generally considered safe, larger scale-rigorous studies are required to confirm their optimal usability as a therapeutic option for neurodegeneration among the elderly.

## 5. Challenges and Potential Solutions to Using Dietary Phytochemicals as Remedy for Age-Related Neurodegenerative Disorders

Generally, dietary phytochemicals have the advantage of being sourced from plant-based foods, offering a cost-effective option with lesser side effects than conventional pharmacological agents [158]. However, adopting dietary phytochemicals as therapeutic agents in the management of neurodegenerative disorders is still limited by several challenges. Currently, most research on the efficacy of dietary phytochemicals is still in the preclinical stages [159]. Although noteworthy results have been recorded in these studies, there is still a need to translate these findings into well-designed clinical trials [110]. In addition, dietary phytochemicals, due to their natural state, have limited stability when compared with synthetic pharmacological agents [160]. As a result of this, they are easily degraded, which reduces their bioavailability and consequently their therapeutic potential [149]. Another major drawback to phytochemical-based therapy is the BBB, which restricts the passage of some natural compounds into their sites of action in the brain [83]. This limits their distribution to target tissues and reduces their efficacy [83].

To implement dietary phytochemical-based therapies for neurodegenerative disorders linked to aging, comprehensive clinical trials are necessary to establish safe and effective dosing regimens [161]. In addition, strategies to improve the bioavailability of phytochemicals and their distribution in neuronal tissues are needed [162]. Nanoparticle-based drug delivery systems offer a potential strategy to enhance the availability and effectiveness of phytochemicals in the brain [163]. Nanocarriers can penetrate the BBB easily and may therefore be utilized as drug transporters for dietary phytochemicals and thus improve the stability, distribution, and therapeutic efficacy of dietary phytochemical-based treatments within the brain tissue [163].

## 6. Conclusions

In the elderly population, neurodegenerative disorders remain a leading cause of morbidity and mortality. Despite extensive research efforts, neurodegenerative disorders remain incurable and barely manageable. Current treatment options offer only limited symptomatic relief without halting disease progression or reversing neuronal loss [9]. In recent times, however, research interest has increased in the potential of dietary phytochemicals as neuroprotective agents due to their antioxidant, anti-inflammatory, and mitochondrial regulating properties [111]. Preclinical studies in cellular and animal models have shown that dietary phytochemicals exert promising effects by influencing changes in key signaling pathways involved in neurodegeneration [158]. However, the bioavailability and clinical efficacy of dietary phytochemicals continue to pose significant barriers to their translation into human and clinical trials [159]. Future research would require the prioritization of well-designed clinical trials and pharmacokinetic studies to establish effective delivery strategies, appropriate dosing, and the therapeutic efficacy of dietary phytochemical-based therapies in human populations [157]. Optimizing phytochemicals and integrating them into preventive therapies may provide a novel approach to address the disease burden of age-related neurodegenerative disorders.

## Figures and Tables

**Table 1:** Side effects and limitations of traditional treatments of neurodegeneration.

Drug Class (Drug)	Mechanism	Side Effects	Limitation	Ref.
ChEIs (donepezil, rivastigmine, and galantamine)	Increased levels of acetylcholine by blocking catalytic action of acetylcholinesterase enzymes	Appetite loss, vomiting, nausea, diarrhea, and rhinitis	Limited efficacy in reducing symptoms of neurodegeneration; no effect on AD progression; usage of ChEIs is associated with several gastrointestinal side effects	[54]
NMDA (memantine)	Strong voltage-dependent but low potency n-methyl-d-aspartate receptor antagonism	Amyloid-related imaging abnormalities	No evidence for slowing down AD progression long-term; memantine is a non-specific NMDA receptor antagonist	[55]
Dopaminergic agents (levodopa)	Dopamine replacement	Motor fluctuations and dyskinesia	Levodopa is linked to long-term motor disorders; requires combination therapy for optimal safety and efficacy	[56]
Antipsychotic drugs (aripiprazole, olanzapine, and risperidone)	Dopaminergic and serotonergic blockage	Extrapyramidal symptoms and adverse metabolic and endocrine effects	Use of antipsychotics is limited by severe adverse effects especially when in combination with other medications for neurodegeneration; requires extensive and intensive drug selection and monitoring	[57,58,59]
Monoclonal antibodies (lecanemab and donanemab)	Microglial activation via Aβ binding	Dizziness, fatigue, sinusitis, upper respiratory tract infections, headaches, orthostatic hypertension, and amyloid-related imaging abnormalities	Not recommended for people with cerebrovascular diseases or ischemic stroke; requires prolonged treatment to achieve meaningful reduction in amyloid plaques	[63,65]
Antisense oligonucleotide (tofersen)	Reduction of dysfunctional SOD1 protein by binding and cleaving *SOD1* mRNA	Pain, fatigue, arthralgia, and myalgia	Intrathecal administration; long-term safety and efficacy are still unknown	[68]

Aβ, amyloid beta; AD, Alzheimer’s disease; ChEI, cholinesterase inhibitor; mRNA, messenger RNA; NMDA, N-methyl-D-aspartate; SOD1, superoxide dismutase 1.

**Table 2:** Dietary phytochemicals targeting aging-related mechanisms in neurodegenerative diseases.

Phytochemical Class	Dietary Phytochemical	Source	Molecular Structure	Experimental Model	Effects	Ref.
Flavone	Apigenin	Celery and parsley	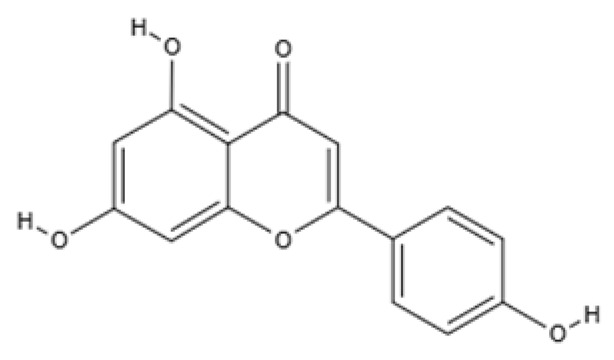	Male C57BL/6N mice	Normalized IFN*γ* expression levels reduced in old mice from approximately 2.0 to 1.0; learning and memory in old mice improved compared with control (*p* < 0.05); recognition index in old mice increased from approximately 40% to 80% compared with controls (*p* < 0.05)	[99]
Phenolic acid	Ferulic acid	Whole grains, fruits, and vegetables	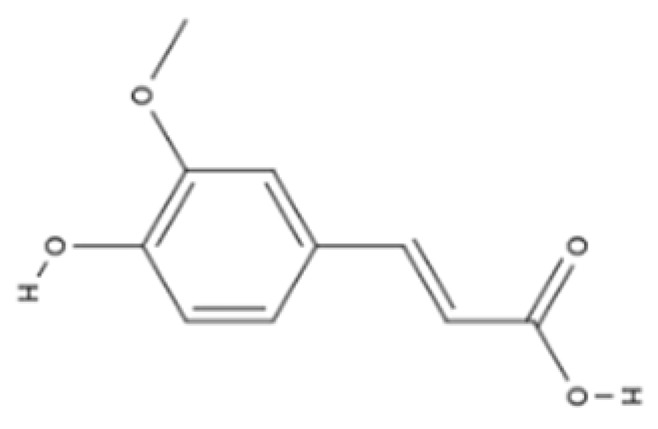	BALB/c mice and HepG2 hepatoma cells	ROS level in cells treated with ferulic acid decreased from approximately 1.6 to 1.25 fluorescence intensity units compared with iron-treated cells; GSH levels increased from approximately 40 nmol/mL to 65 nmol/mL compared with iron-treated cells	[100]
Flavonol	Quercetin	Fruits and vegetables	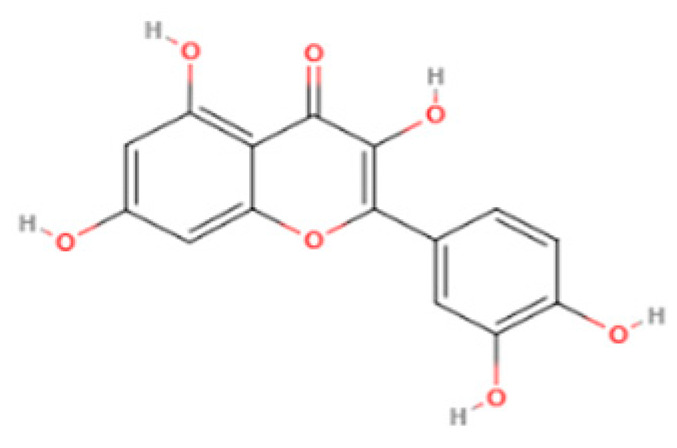	Male Wistar rats treated with 6-OHDA	Grip strength improved from approximately 0.53 to 0.75 KgF; inflammatory biomarkers were reduced: TNF-α from approximately 200 to 175 pg/mg, IL-1β from approximately 175 to 100 pg/mg, IL-6 from approximately 212.5 to 137.5 pg/mg	[101]
Isoflavone	Genistein	Soy and soy-derived products	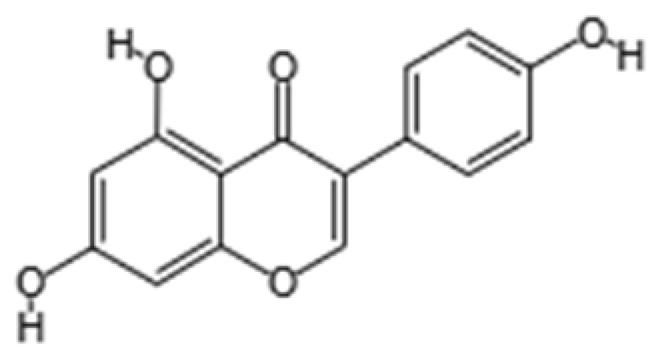	*Caenorhabditis elegans*	Mean survival rate of oxidative-stress-exposed *C. elegans* increased by 56.7%; lipofuscin and ROS accumulation reduced by 44.4% and 47.9% respectively; SOD activity increased by 67.5% in H_2_O_2_-treated worms	[102]
Flavanol	Fisetin	Apples, berries, and vegetables	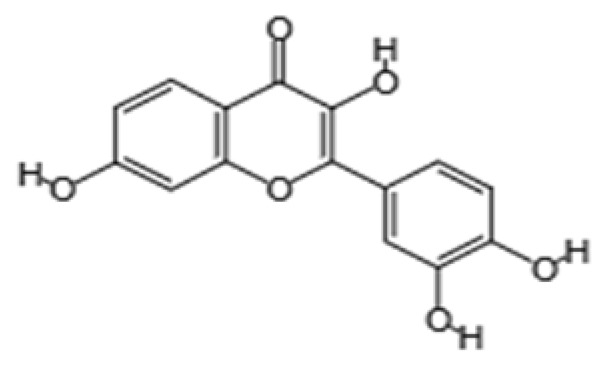	Male C57BL/6J mice	Upregulation of aging markers was suppressed: relative mRNA expression of CDKN1A decreased from 3.5 to 0.75 (*p* = 0.018), and relative mRNA expression of CDKN2A decreased from approximately 5 to 1 (*p* = 0.019)	[103]
Stilbene	Resveratrol	Grapes, peanuts, and red wine	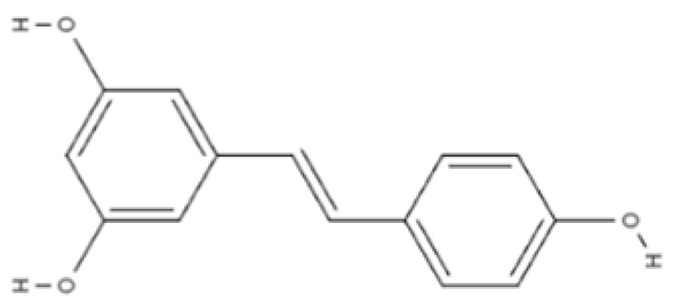	In vitro N9 microglial cell line OGD/R injury model and in vitro HT22 hippocampal neuronal cell line	SOD levels increased from approximately 27 u/mL to 45 u/mL; MDA levels reduced from 0.8 to 0.6 nmol/mL; mitochondrial function improved via activation of the Nrf2 pathway	[104]
Nitrogen-containing phytochemical	Tomatidine	Tomatoes	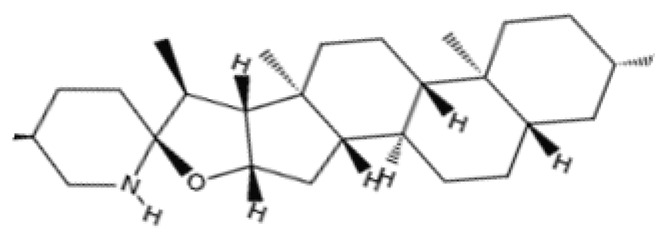	*Caenorhabditis elegans*	Mitochondrial network morphology score increased from 3.4 in control to 3.9; mt-mKeima signal increased to 561 nm; survival increased by 19%	[105]
Carotenoid	Lycopene	Tomatoes, guava, and watermelon	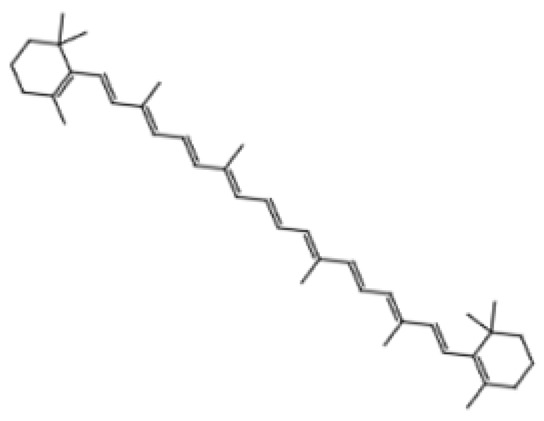	Human SH-SY5Y neuroblastoma cells	Percentage of cells with apoptotic features decreased from 32.6 ± 4.8% to 25.1 ± 1.9% and 15.2 ± 1.7% (mean ± SD) when treated with 2.0 and 4.0 µmol/l lycopene respectively; apoptosis rate reduced from 42.04% to 26.55% and 17.87% when treated with 2.0 and 4.0 µmol/l lycopene, respectively; lycopene (2.0 and 4.0 µmol/l) increased mitochondrial membrane potentials by 8.71% and 16.42%, respectively	[106]
Glucosinolate	Glucoraphanin	Cruciferous vegetables	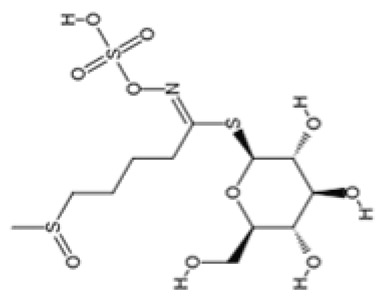	C57BL/6 mice	Relative Nrf2 protein content improved from approximately 1 to 1.125 (*p* < 0.05); mitochondrial biogenesis increased; macrophage infiltration was inhibited	[107]
Flavanone	Hesperidin	Citrus fruits	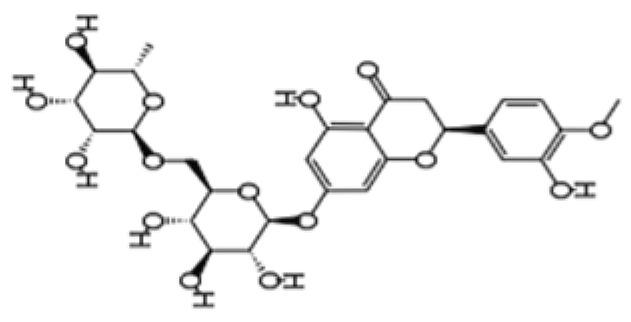	Lewis rats	Compared with control: IEL TCRγδ^+^ cells increased to 140%, CD45RA^+^ increased to 180%, TCRαβ^+^CD4^+^ increased to 132%; LPL TCRγδ^+^ decreased to 35%, NK cells decreased to 29%, TCRαβ^+^CD8^+^ decreased to 52%, NKT cells decreased to 42%, CD4^+^CD103^+^ decreased to 50%, and CD8^+^CD103^+^ decreased to 60%	[108]
Lignan	Pinoresinol	Flax seeds and sesame seeds	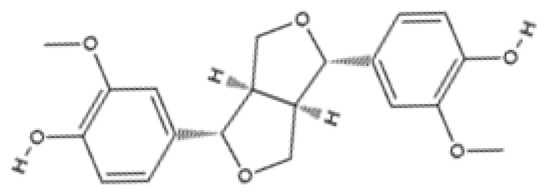	Human epithelial breast cells (MDA-MB-231 and MCF7)	Weak antioxidant activity (DPPH assay) was observed: 50% RSA at 69 μM compared with 11 μM for α-tocopherol (control)	[109]

BALB/c mice, Bagg albino mice; C57BL/6J, Jackson Laboratory substrain of C57BL/6 inbred mouse; C57BL/6N, National Institute of Health substrain of C57BL/6 inbred mouse; CD103*^+^*, integrin αE positive mucosa-resident T cells; CD4*^+^*, T helper cells; CD45RA*^+^*, naïve B cells; CD8*^+^*, cytotoxic T cells; CDKN1A, cyclin-dependent kinase inhibitor 1A; CDKN2A, cyclin-dependent kinase inhibitor 2A; DNA, deoxyribonucleic acid; DPPH, 2,2-diphenyl-1-picrylhydrazyl; GSH, reduced glutathione; H_2_O_2_, hydrogen peroxide; HepG2, human hepatocellular carcinoma cell line; IEL, intraepithelial lymphocytes; IFNγ, interferon gamma; IL-1β, interleukin-1 beta; IL-6, interleukin-6; KgF, kilogram force; LPL, lamina propria lymphocytes; MCF7, Michigan Cancer Foundation-7 human breast cancer cell line; MDA, malonaldehyde; MDA-MB-231, M.D. Anderson-Metastatic Breast-231 human breast cancer cell line; mt-Keima, mitochondria-targeted Keima; N9, mouse microglial cell line; NK cells, natural killer cells; NKT cells, natural killer T cells; Nrf2,nuclear factor erythroid 2-related factor 2; OGD/R, oxygen-glucose deprivation/reoxygenation; 6-OHDA, 6-hydroxydopamine; ROS, reactive oxygen species; RSA, radical scavenging activity; SH-SY5Y, human neuroblastoma cell line; SOD, superoxide dismutase; TCRαβ*^+^*, T-cell receptor alpha-beta positive cells; TCRγδ*^+^*, T-cell receptor gamma-delta positive cells; TNF-α, tumor necrosis factor alpha. Recognition index was converted from ratios to percentages for clarity. Approximate values were estimated from graphs where exact data are not reported.

**Table 3:** Effectiveness of dietary phytochemicals targeting aging hallmarks to alleviate neurodegeneration: preclinical studies.

Phytochemical	Neurodegenerative Disorder	Experimental Model	Aging Hallmarks Targeted	Effects	Ref.
Berberine	AD	Transgenic APP/tau/PS1 mice	Macroautophagy and disabled proteostasis	Memory impairment was reduced; Aβ concentrations reduced from approximately 850 μg/L to 600 μg/L levels	[125]
Baicalein	AD	Transgenic APP/PS1 mice	Dysbiosis	Recognition index improved by about 20% (*p* < 0.01); gut microbiota was dominated by Bacteroidetes (14.59–67.02%) and Firmicutes (20.19–61.39%).	[126]
Resveratrol	ALS	Transgenic SOD1^G93A^ ALS mice	Mitochondrial dysfunction, inflammaion	Microglial reactivity reduced from over 1152% to 649%; motor neuron counts increased from 18.2 ± 1.3 (mean ± SEM) in untreated mice to 35.2 ± 1.1 (mean ± SEM) in resveratrol-treated mice	[127]
	PD	Male Sprague–Drawley rats and rotenone-induced PC12 cell line	Mitochondrial dysfunction	Improved mitochondrial mass, homeostasis, and neuronal function; ROS fluorescence reduced from approximately 175 to 115 (*p* < 0.05); ATP concentration (relative to control) increased from approximately 25% to 60% (*p* < 0.05)	[128]
Quercetin	HTTD	Female Wistar rats	Mitochondrial dyfunction	Mitochondrial function increased by 39.3%; total mitochondrial thiols increased by 37.5%; locomotor function improved from 80 to 150 counts; gait abnormalities were prevented	[129]
Curcumin	AD	APP/PS1 transgenic mice	Stem cell exhaustion	Neuronal stem cell proliferation was activated; neurogenesis improved; water maze latency reduced from 37.6 s in controls to 27.3 s in treated mice; hippocampal neuronal apoptosis count decreased from 40 in controls to 30 in treated mice	[130]
	AD	Transgenic AD mouse model	Genomic instability and telomere attrition	Telomere length increased from 0.47 ± 0.70 kb (mean ± SD) in AD control mice to 1.25 ± 3.15 kb (mean ± SD) in treated mice	[131]

Aβ, amyloid beta; AD, Alzheimer’s disease; ALS, amyotrophic lateral sclerosis; APP, amyloid precursor protein; ATP, Adenosine triphosphate; HTTD, Huntington’s disease; MN, motor neuron; MTT, (4,5-dimethylthiazol-2-yl)-2,5-diphenyltetrazolium bromide; PC12, rat pheochromocytoma cell line; PD, Parkinson’s disease; PS1, mutated presenilin 1; ROS, reactive oxygen species; SD, standard deviation; SEM, standard error of the mean; SOD1^G93A^, human superoxide dismutase-1 with G93A mutation; TUNEL, terminal deoxynucleotidyl transferase dUTP nick-end labeling. Approximate values were estimated from graphs where exact data are not reported.

**Table 4:** Effectiveness of dietary phytochemicals targeting aging hallmarks to alleviate neurodegeneration: clinical trials.

Phytochemical	Source	Neurodegenerative Disorder	Study Design	Dose and Duration	Mechanism Targeted	Effects	Ref.
Mucuna pruriens powder	*Mucuna pruriens*	PD	Non-inferiority, phase 2b randomized, double-blind, controlled crossover study (N = 18)	17.5 mg/kg (high dose), 12.5 mg/kg (low dose), 3.5 mg/kg (with benserazide) for 6 days	Dopamine deficiency	Motor performance improved with MP at 90 min (*p* = 0.037) and 180 min (*p* = 0.002); UPDRS-III scores decreased by 16% with MP-LD and by 50% with MP + DDCI at 180 min; mean ‘on’ time increased to 221 min for MP-HD compared with 177 min for LD-DCI (*p* < 0.001)	[132]
Curcumin (unformulated standard curcumin (USC) and curcumin–galactomannan complex (CGM))	Turmeric	Dementia	Three-arm, randomized, double-blind, parallel-group clinical trial (N = 48)	400 mg/day twice daily for six months	Inflammation, neurotrophic modulation, proteostasis	Locomotive function increased with CGM compared with placebo (F = 59.95, *p* = 0.001); GLFS-25 scores decreased by 25.7% from baseline; Aβ42 levels decreased by 23.3% compared with placebo and 16.0% compared with USC; tau proteins reduced by 22.8%	[133]
Quercetin	Fruits and vegetables	Mild AD	Single-arm, interventional feasibility study (N = 12)	100 mg dasatinib + 1250 mg quercetin (DQ) for 2 days every 2 weeks for a total of 12 weeks	Senescence	DQ increased MoCA scores by 2 points (95% CI: 0.1–4.0) in participants with lowest baseline scores	[134]
Genistein	Soy and soy-derived products	AD	Double-blind, placebo-controlled bicentric study (N = 27)	120 mg for 12 months	Proteostasis	Genistein treatment improved cognitive tests (*p* = 0.002) and stabilized Aβ levels	[135]
Resveratrol	Grapes, berries, and peanuts	Mild cognitive impairment	Randomized, double-blind interventional study (N = 40)	200 mg/day for 26 weeks	Nutrient sensing and neural connectivity	HbA1c reduced by 0.15% (*t*_(17)_ = 3.3, *p* = 0.005, Cohen’s *d* = 1.60); hippocampal volume was preserved; functional connectivity improved between the right anterior hippocampus and right angular cortex	[136]
		AD	Retrospective study (N = 119)	500 mg dose once daily, increasing by 500 mg every 13 weeks to 1000 mg twice daily	Neuroinflammation	Decline in cognitive test scores was reduced (*p* < 0.0001); CSF MMP-9 levels decreased by 48%	[137]
Ginkgo extract	Ginkgo biloba	VaD	Double-blind, randomized, controlled study (N = 196)	1 tablet of gingko extract thrice daily and 4.8 g of shenmayizhi formula twice daily for 12 weeks	Vascular endothelial function	Vascular endothelial functions improved compared with control (*p* < 0.05)	[138]

Aβ, amyloid-beta; AD, Alzheimer’s disease; CGM, curcumin–galactomannan complex; CSF, cerebrospinal fluid; DDCI, dopa-decarboxylase inhibitor; DQ, 100 mg dasatinib + 1250 mg quercetin; GLFS-25, 25-question Geriartric Locomotive Function Scale; hbA1c, glycated hemoglobin; MMP-9, matrix metalloproteinase-9; MoCA, Montreal Cognitive Assessment; MP, mucuna puriens; MP-LD, low dose mucuna puriens powder; MP-HD, high dose mucuna puriens powder; N, number of participants; PD, Parkinson’s disease; TNF-α, tumor necrosis factor alpha; UPDRS-III, Unified Parkinson’s Disease Rating Scale Part III; USC, unformulated standard curcumin; VaD, vascular dementia. Approximate values were estimated from graphs where exact data are not reported.

## References

[1] Cheng X., Yang Y., Schwebel D.C., Liu Z., Li L., Cheng P., Ning P., Hu G. (2020). Population ageing and mortality during 1990–2017: A global decomposition analysis. PLoS Med..

[2] Yang G., Zhang L. (2023). Relationship between aging population, birth rate and disposable income per capita in the context of COVID-19. PLoS ONE.

[3] GBD 2021 Causes of Death Collaborators (2024). Global burden of 288 causes of death and life expectancy decomposition in 204 countries and territories and 811 subnational locations, 1990–2021: A systematic analysis for the Global Burden of Disease Study 2021. Lancet.

[4] Khan H.T., Addo K.M., Findlay H. (2024). Public health challenges and responses to the growing ageing populations. Public Health Chall..

[5] Montégut L., López-Otín C., Kroemer G. (2024). Aging and cancer. Mol. Cancer.

[6] Bacanoiu M.V., Danoiu M. (2022). New strategies to improve the quality of life for normal aging versus pathological aging. J. Clin. Med..

[7] Nabi M., Tabassum N. (2022). Role of environmental toxicants on neurodegenerative disorders. Front. Toxicol..

[8] Wimo A., Seeher K., Cataldi R., Cyhlarova E., Dielemann J.L., Frisell O., Guerchet M., Jönsson L., Malaha A.K., Nichols E. (2023). The worldwide costs of dementia in 2019. Alzheimer Dement..

[9] Palanisamy C.P., Pei J., Alugoju P., Anthikapalli N.V.A., Jayaraman S., Veeraraghavan V.P., Gopathy S., Roy J.R., Janaki C.S., Thalamati D. (2023). New strategies of neurodegenerative disease treatment with extracellular vesicles (EVs) derived from mesenchymal stem cells (MSCs). Theranostics.

[10] Xavier J.R., Sameer B., Gupta D., Mehta S., Chauhan O.P. (2024). Bioactive compounds of foods: Phytochemicals and peptides. Food Hum..

[11] Manach C., Scalbert A., Morand C., Rémésy C., Jiménez L. (2004). Polyphenols: Food sources and bioavailability. Am. J. Clin. Nutr..

[12] Muscolo A., Mariateresa O., Giulio T., Mariateresa R. (2024). Oxidative stress: The role of antioxidant phytochemicals in the prevention and treatment of diseases. Int. J. Mol. Sci..

[13] Jiang Q., Liu J., Huang S., Wang X.-Y., Chen X., Liu G.-H., Ye K., Song W., Masters C.L., Wang J. (2025). Antiageing strategy for neurodegenerative diseases: From mechanisms to clinical advances. Signal Transduct. Target. Ther..

[14] Griñán-Ferré C., Bellver-Sanchis A., Guerrero A., Pallàs M. (2024). Advancing personalized medicine in neurodegenerative diseases: The role of epigenetics and pharmacoepigenomics in pharmacotherapy. Pharmacol. Res..

[15] Solovev I., Sergeeva A., Geraskina A., Shaposhnikov M., Vedunova M., Borysova O., Moskalev A. (2024). Aging and physiological barriers: Mechanisms of barrier integrity changes and implications for age-related diseases. Mol. Biol. Rep..

[16] Buttari B., Tramutola A., Rojo A.I., Chondrogianni N., Saha S., Berry A., Giona L., Miranda J.P., Profumo E., Davinelli S. (2025). Proteostasis Decline and Redox Imbalance in Age-Related Diseases: The Therapeutic Potential of NRF2. Biomolecules.

[17] López-Otín C., Blasco M.A., Partridge L., Serrano M., Kroemer G. (2023). Hallmarks of aging: An expanding universe. Cell.

[18] López-Otín C., Blasco M.A., Partridge L., Serrano M., Kroemer G. (2013). The hallmarks of aging. Cell.

[19] Huang R., Zhou P.-K. (2021). DNA damage repair: Historical perspectives, mechanistic pathways and clinical translation for targeted cancer therapy. Signal Transduct. Target. Ther..

[20] Chandimali N., Bak S.G., Park E.H., Lim H.-J., Won Y.-S., Kim E.-K., Park S.-I., Lee S.J. (2025). Free radicals and their impact on health and antioxidant defenses: A review. Cell Death Discov..

[21] Shadfar S., Parakh S., Jamali M.S., Atkin J.D. (2023). Redox dysregulation as a driver for DNA damage and its relationship to neurodegenerative diseases. Transl. Neurodegener..

[22] Li Y., Tian X., Luo J., Bao T., Wang S., Wu X. (2024). Molecular mechanisms of aging and anti-aging strategies. Cell Commun. Signal..

[23] Meaza I., Cahill C.R., Speer R.M., Kouokam J.C., Wise J.P. (2025). Particulate hexavalent chromium inhibits global transcription of genes in DNA repair pathways, particularly targeting homologous recombination repair, base excision repair, mismatch repair and microhomology-mediated end-joining. J. Hazard. Mater..

[24] Schumacher B., Pothof J., Vijg J., Hoeijmakers J.H.J. (2021). The central role of DNA damage in the ageing process. Nature.

[25] Panier S., Wang S., Schumacher B. (2024). Genome instability and DNA repair in somatic and reproductive aging. Annu. Rev. Pathol..

[26] Lu X., Liu L. (2024). Genome stability from the perspective of telomere length. Trends Genet..

[27] Wu Z., Qu J., Liu G.-H. (2024). Roles of chromatin and genome instability in cellular senescence and their relevance to ageing and related diseases. Nat. Rev. Mol. Cell Biol..

[28] Wang K., Liu H., Hu Q., Wang L., Liu J., Zheng Z., Zhang W., Ren J., Zhu F., Liu G.-H. (2022). Epigenetic regulation of aging: Implications for interventions of aging and diseases. Sig. Trans. Target. Ther..

[29] Hadad N., Masser D.R., Blanco-Berdugo L., Stanford D.R., Freeman W.M. (2019). Early-life DNA methylation profiles are indicative of age-related transcriptome changes. Epigenetics Chromatin.

[30] Lettieri-Barbato D., Aquilano K., Punziano C., Minopoli G., Faraonio R. (2022). MicroRNAs, long non-coding RNAs, and circular RNAs in the redox control of cell senescence. Antioxidants.

[31] Liu Y., Tan Y., Zhang Z., Yi M., Zhu L., Peng W. (2024). The interaction between ageing and Alzheimer’s disease: Insights from the hallmarks of ageing. Transl. Neurodegener..

[32] Gómez-Virgilio L., Silva-Lucero M.-D.-C., Flores-Morelos D.-S., Gallardo-Nieto J., Lopez-Toledo G., Abarca-Fernandez A.-M., Zacapala-Gómez A.-E., Luna-Muñoz J., Montiel-Sosa F., Soto-Rojas L.O. (2022). Autophagy: A key regulator of homeostasis and disease: An overview of molecular mechanisms and modulators. Cells.

[33] Lim S.H., Hansen M., Kumsta C. (2024). Molecular mechanisms of autophagy decline during aging. Cells.

[34] Varela-Eirín M., Demaria M. (2022). Cellular senescence. Curr. Biol..

[35] Dou X., Fu Q., Long Q., Liu S., Zou Y., Fu D., Xu Q., Jiang Z., Ren X., Zhang G. (2023). PDK4-dependent hypercatabolism and lactate production of senescent cells promotes cancer malignancy. Nat. Metab..

[36] Miwa S., Kashyap S., Chini E., von Zglinicki T. (2022). Mitochondrial dysfunction in cell senescence and aging. J. Clin. Investig..

[37] Ruggiero C., Tafaro L., Cianferotti L., Tramontana F., Macchione I.G., Caffarelli C., Virdis A., Ferracci M., Rinonapoli G., Mecocci P. (2024). Targeting the Hallmarks of Aging with Vitamin D: Starting to Decode the Myth. Nutrients.

[38] Molinero N., Antón-Fernández A., Hernández F., Ávila J., Bartolomé B., Moreno-Arribas M.V. (2023). Gut microbiota, an additional hallmark of human aging and neurodegeneration. Neuroscience.

[39] Ji S., Xiong M., Chen H., Liu Y., Zhou L., Hong Y., Wang M., Wang C., Fu X., Sun X. (2023). Cellular rejuvenation: Molecular mechanisms and potential therapeutic interventions for diseases. Signal Transduct. Target. Ther..

[40] Sachdev P.S., Blacker D., Blazer D.G., Ganguli M., Jeste D.V., Paulsen J.S., Petersen R.C. (2014). Classifying neurocognitive disorders: The DSM-5 approach. Nat. Rev. Neurol..

[41] Cipriani G., Danti S., Picchi L., Nuti A., Fiorino M.D. (2020). Daily functioning and dementia. Dement. Neuropsychol..

[42] Sharifi F., Ghandali R., Alimohammadi M., Ahmadipour P., Moradikor N., Chatterjee I., Mohamed W. (2024). Symptoms and diagnosis of dementia. Nutrition in Brain Aging and Dementia.

[43] Bir S.C., Khan M.W., Javalkar V., Toledo E.G., Kelley R.E. (2021). Emerging concepts in vascular dementia: A review. J. Stroke Cerebrovasc. Dis..

[44] Kamatham P.T., Shukla R., Khatri D.K., Vora L.K. (2024). Pathogenesis, diagnostics, and therapeutics for Alzheimer’s disease: Breaking the memory barrier. Ageing Res. Rev..

[45] Simon C., Soga T., Okano H.J., Parhar I. (2021). α-Synuclein-mediated neurodegeneration in Dementia with Lewy bodies: The pathobiology of a paradox. Cell Biosci..

[46] Latif S., Jahangeer M., Razia D.M., Ashiq M., Ghaffar A., Akram M., Allam A.E., Bouyahya A., Garipova L., Shariati M.A. (2021). Dopamine in Parkinson’s disease. Clin. Chim. Acta..

[47] Wang Q., Liu Y., Zhou J. (2015). Neuroinflammation in Parkinson’s disease and its potential as therapeutic target. Transl. Neurodegener..

[48] Salim S., Ahmad F., Banu A., Mohammad F. (2023). Gut microbiome and Parkinson’s disease: Perspective on pathogenesis and treatment. J. Adv. Res..

[49] Yedavalli V.S., Patil A., Shah P. (2018). Amyotrophic Lateral Sclerosis and its Mimics/Variants: A Comprehensive Review. J. Clin. Imaging Sci..

[50] Dashtmian A.R., Darvishi F.B., Arnold W.D. (2024). Chronological and Biological Aging in Amyotrophic Lateral Sclerosis and the Potential of Senolytic Therapies. Cells.

[51] Machiela E., Southwell A.L. (2020). Biological aging and the Cellular Pathogenesis of Huntington’s Disease. J. Huntingtons Dis..

[52] Ihunwo A.O., Tembo L.H., Dzamalala C. (2016). The dynamics of adult neurogenesis in human hippocampus. Neural Regen. Res..

[53] Singh K., Gupta J.K., Kumar S., Soni U. (2024). A Review of the Common Neurodegenerative Disorders: Current Therapeutic Approaches and the Potential Role of Bioactive Peptides. Curr. Protein Pept. Sci..

[54] Moreira N., Lima J., Marchiori M.F., Carvalho I., Sakamoto-Hojo E.T. (2022). Neuroprotective Effects of Cholinesterase Inhibitors: Current Scenario in Therapies for Alzheimer’s Disease and Future Perspectives. J. Alzheimer’s Dis. Rep..

[55] Tari P.K., Parsons C.G., Collingridge G.L., Rammes G. (2024). Memantine: Updating a rare success story in pro-cognitive therapeutics. Neuropharmacology.

[56] Zhuo C., Zhu X., Jiang R., Ji F., Su Z., Xue R., Zhou Y. (2017). Comparison for Efficacy and Tolerability among Ten Drugs for Treatment of Parkinson’s Disease: A Network Meta-Analysis. Sci. Rep..

[57] Rybakowski J.K. (2023). Application of Antipsychotic Drugs in Mood Disorders. Brain Sci..

[58] Gurevich A., Guller V., Berner Y.N., Tal S. (2012). Are atypical antipsychotics safer than typical antipsychotics for treating behavioral and psychological symptoms of dementia?. J. Nutr. Health Aging.

[59] Ohno Y., Kunisawa N., Shimizu S. (2019). Antipsychotic Treatment of Behavioral and Psychological Symptoms of Dementia (BPSD): Management of Extrapyramidal Side Effects. Front. Pharmacol..

[60] Durães F., Pinto M., Sousa E. (2018). Old Drugs as New Treatments for Neurodegenerative Diseases. Pharmaceuticals.

[61] Niidome T., Ishikawa Y., Ogawa T., Nakagawa M., Nakamura Y. (2024). Mechanism of action and clinical trial results of Lecanemab (Leqembi(®) 200 mg, 500 mg for Intravenous Infusion), a novel treatment for Alzheimer’s disease. Nihon Yakurigaku Zasshi.

[62] Ono K., Tsuji M. (2020). Protofibrils of Amyloid-β are Important Targets of a Disease-Modifying Approach for Alzheimer’s Disease. Int. J. Mol. Sci..

[63] Chowdhury S., Chowdhury N.S. (2023). Novel anti-amyloid-beta (Aβ) monoclonal antibody lecanemab for Alzheimer’s disease: A systematic review. Int. J. Immunopathol. Pharmacol..

[64] Zhang Y., Chen H., Li R., Sterling K., Song W. (2023). Amyloid β-based therapy for Alzheimer’s disease: Challenges, successes and future. Sig. Transduct. Target. Ther..

[65] Jayaprakash N., Elumalai K. (2024). Translational Medicine in Alzheimer’s Disease: The Journey of Donanemab from Discovery to Clinical Application. Chronic Dis. Transl. Med..

[66] Power D., Crotty G.F. (2025). Advances in the Pharmacological Management of Parkinson’s Disease. Curr. Treat. Options Neurol..

[67] Carbone F., Djamshidian A., Seppi K., Poewe W. (2019). Apomorphine for Parkinson’s Disease: Efficacy and Safety of Current and New Formulations. CNS Drugs.

[68] Jin J., Zhong X.B. (2023). ASO drug Qalsody (tofersen) targets amyotrophic lateral sclerosis. Trends Pharmacol. Sci..

[69] Kim A.Y., Al Jerdi S., MacDonald R., Triggle C.R. (2024). Alzheimer’s disease and its treatment-yesterday, today, and tomorrow. Front. Pharmacol..

[70] Raza M.L., Bhojani A., Batool S.K., Zehra D. (2024). Non pharmacological approaches for neurodegenerative diseases: A narrative review. Exp. Gerontol..

[71] Hu J., Huang B., Chen K. (2024). The impact of physical exercise on neuroinflammation mechanism in Alzheimer’s disease. Front. Aging Neurosci..

[72] He Y., Wang Q., Wu H., Dong Y., Peng Z., Guo X., Jiang N. (2023). The role of IGF-1 in exercise to improve obesity-related cognitive dysfunction. Front. Neurosci..

[73] Martin B., Mattson M.P., Maudsley S. (2006). Caloric restriction and intermittent fasting: Two potential diets for successful brain aging. Ageing Res. Rev..

[74] Sun J., Roy S. (2021). Gene-based therapies for neurodegenerative diseases. Nat. Neurosci..

[75] Sivandzade F., Cucullo L. (2021). Regenerative Stem Cell Therapy for Neurodegenerative Diseases: An Overview. Int. J. Mol. Sci..

[76] Li J., Li N., Wei J., Feng C., Chen Y., Chen T., Ai Z., Zhu X., Ji W., Li T. (2022). Genetically engineered mesenchymal stem cells with dopamine synthesis for Parkinson’s disease in animal models. NPJ Park. Dis..

[77] Cha M.Y., Kwon Y.W., Ahn H.S., Jeong H., Lee Y.Y., Moon M., Baik S.H., Kim D.K., Song H., Yi E.C. (2017). Protein-Induced Pluripotent Stem Cells Ameliorate Cognitive Dysfunction and Reduce Aβ Deposition in a Mouse Model of Alzheimer’s Disease. Stem Cells Transl. Med..

[78] Santiago J.A., Potashkin J.A. (2023). Physical activity and lifestyle modifications in the treatment of neurodegenerative diseases. Front. Aging Neurosci..

[79] Fritz N.E., Rao A.K., Kegelmeyer D., Kloos A., Busse M., Hartel L., Carrier J., Quinn L. (2017). Physical Therapy and Exercise Interventions in Huntington’s Disease: A Mixed Methods Systematic Review. J. Huntingt. Dis..

[80] Erkkinen M.G., Kim M.O., Geschwind M.D. (2018). Clinical Neurology and Epidemiology of the Major Neurodegenerative Diseases. Cold Spring Harb. Perspect. Biol..

[81] Kwon D.K., Kwatra M., Wang J., Ko H.S. (2022). Levodopa-Induced Dyskinesia in Parkinson’s Disease: Pathogenesis and Emerging Treatment Strategies. Cells.

[82] Qian L., He X., Liu Y., Gao F., Lu W., Fan Y., Gao Y., Wang W., Zhu F., Wang Y. (2023). Longitudinal Gut Microbiota Dysbiosis Underlies Olanzapine-Induced Weight Gain. Microbiol. Spectr..

[83] Wu D., Chen Q., Chen X., Han F., Chen Z., Wang Y. (2023). The blood–brain barrier: Structure, regulation and drug delivery. Sig. Transduct. Target. Ther..

[84] de Carvalho T.S. (2022). Calorie restriction or dietary restriction: How far they can protect the brain against neurodegenerative diseases?. Neural Regen. Res..

[85] Rabizadeh F., Mirian M.S., Doosti R., Kiani-Anbouhi R., Eftekhari E. (2022). Phytochemical classification of medicinal plants used in the treatment of kidney disease based on traditional Persian medicine. Evid. Based. Complement. Alternat. Med..

[86] Pandey K.B., Rizvi S.I. (2009). Plant polyphenols as dietary antioxidants in human health and disease. Oxid. Med. Cell Longev..

[87] Hossain M.S., Wazed M.A., Asha S., Amin M.R., Shimul I.M. (2025). Dietary Phytochemicals in Health and Disease: Mechanisms, Clinical Evidence, and Applications—A Comprehensive Review. Food Sci. Nutr..

[88] Jalouli M., Rahman M.A., Biswas P., Rahman H., Harrath A.H., Lee I.-S., Kang S., Choi J., Park M.N., Kim B. (2025). Targeting natural antioxidant polyphenols to protect neuroinflammation and neurodegenerative diseases: A comprehensive review. Front. Pharmacol..

[89] Rathod N.B., Elabed N., Punia S., Ozogul F., Kim S.K., Rocha J.M. (2023). Recent Developments in Polyphenol Applications on Human Health: A Review with Current Knowledge. Plants.

[90] Shanaida M., Mykhailenko O., Lysiuk R., Hudz N., Balwierz R., Shulhai A., Shapovalova N., Shanaida V., Bjørklund G. (2025). Carotenoids for Antiaging: Nutraceutical, Pharmaceutical, and Cosmeceutical Applications. Pharmaceuticals.

[91] Maoka T. (2020). Carotenoids as natural functional pigments. J. Nat. Med..

[92] Connolly E.L., Sim M., Travica N., Marx W., Beasy G., Lynch G.S., Bondonno C.P., Lewis J.R., Hodgson J.M., Blekkenhorst L.C. (2021). Glucosinolates from cruciferous vegetables and their potential role in chronic disease: Investigating the preclinical and clinical evidence. Front. Pharmacol..

[93] Yang W., Chen X., Li Y., Guo S., Wang Z., Yu X. (2020). Advances in pharmacological activities of terpenoids. Nat. Prod. Commun..

[94] Egbujor M.C., Petrosino M., Zuhra K., Saso L. (2022). The role of organosulfur compounds as Nrf2 activators and their antioxidant effects. Antioxidants.

[95] Naik R.A., Rajpoot R., Koiri R.K., Bhardwaj R., Aldairi A.F., Johargy A.K., Faidah H., Babalghith A.O., Hjazi A., Alsanie W.F. (2025). Dietary supplementation and the role of phytochemicals against the Alzheimer’s disease: Focus on polyphenolic compounds. J. Prev. Alzheimers Dis..

[96] Guo J., Huang X., Duo L., Yan M., Shen T., Tang W., Li J. (2022). Aging and aging-related diseases: From molecular mechanisms to interventions and treatments. Sig. Trans. Target. Ther..

[97] Tenchov R., Sasso J.M., Wang X., Zhou Q.A. (2024). Antiaging Strategies and Remedies: A Landscape of Research Progress and Promise. ACS Chem. Neurosci..

[98] Wang Y., Cao X., Ma J., Liu S., Jin X., Liu B. (2024). Unveiling the Longevity Potential of Natural Phytochemicals: A Comprehensive Review of Active Ingredients in Dietary Plants and Herbs. J. Agric. Food Chem..

[99] Cavalier A.N., Clayton Z.S., Wahl D., Hutton D.A., McEntee C.M., Seals D.R., LaRocca T.J. (2024). Protective effects of apigenin on the brain transcriptome with aging. Mech. Ageing Dev..

[100] Kose T., Moreno-Fernandez J., Vera-Aviles M., Sharp P.A., Latunde-Dada G.O. (2023). Ferulic acid protects HepG2 cells and mouse liver from iron-induced damage. Biochem. Biophys. Rep..

[101] Singh S., Kumar P. (2018). Piperine in combination with quercetin halt 6-OHDA induced neurodegeneration in experimental rats: Biochemical and neurochemical evidences. Neurosci. Res..

[102] Zhang S.-Y., Qin Z.-C., Sun Y.-Y., Chen Y.-S., Chen W.-B., Wang H.-G., An D., Sun D., Liu Y.-Q. (2023). Genistein Promotes Anti-Heat Stress and Antioxidant Effects via the Coordinated Regulation of IIS, HSP, MAPK, DR, and Mitochondrial Pathways in Caenorhabditis elegans. Antioxidants.

[103] Zhao R., Kou H., Jiang D., Wang F. (2023). Exploring the anti-aging effects of fisetin in telomerase-deficient progeria mouse model. Peer J..

[104] Liu J., Liao H., Chen Y., Zhu H., Li X., Liu J., Xiang Q., Zeng F., Yang Q. (2022). Resveratrol Inhibits Oxidative Stress and Regulates M1/M2-Type Polarization of Microglia via Mediation of the Nrf2/Shh Signaling Cascade after OGD/R Injury In Vitro. J. Pers. Med..

[105] Fang E.F., Waltz T.B., Kassahun H., Lu Q., Kerr J.S., Morevati M., Fivenson E.M., Wollman B.N., Marosi K., Wilson M.A. (2017). Tomatidine enhances lifespan and healthspan in C. elegans through mitophagy induction via the SKN-1/Nrf2 pathway. Sci. Rep..

[106] Feng C., Luo T., Zhang S., Liu K., Zhang Y., Luo Y., Ge P. (2016). Lycopene protects human SH-SY5Y neuroblastoma cells against hydrogen peroxide-induced death via inhibition of oxidative stress and mitochondria-associated apoptotic pathways. Mol. Med. Rep..

[107] Tian Q., Xu Z., Sun Q., Iniguez A.B., Du M., Zhu M.-J. (2022). Broccoli-Derived Glucoraphanin Activates AMPK/PGC1α/NRF2 Pathway and Ameliorates Dextran-Sulphate-Sodium-Induced Colitis in Mice. Antioxidants.

[108] Camps-Bossacoma M., Franch À., Pérez-Cano F.J., Castell M. (2017). Influence of Hesperidin on the Systemic and Intestinal Rat Immune Response. Nutrients.

[109] López-Biedma A., Sánchez-Quesada C., Beltrán G., Delgado-Rodríguez M., Gaforio J.J. (2016). Phytoestrogen (+)-pinoresinol exerts antitumor activity in breast cancer cells with different oestrogen receptor statuses. BMC Complement. Altern. Med..

[110] Hikmawati V.F., Alam F.M., Ainnayah J.S., Fatchiyah F. (2020). Virtual prediction of phenolic and glucosinolate compounds with Keap1 protein as anti-aging by stimulating Nrf2. J. Exp. Life Sci..

[111] Kim Y., Lim J., Oh J. (2024). Taming neuroinflammation in Alzheimer’s disease: The protective role of phytochemicals through the gut−brain axis. Biomed. Pharmacother..

[112] Boronat A., Rodriguez-Morató J., Serreli G., Fitó M., Tyndale R.F., Deiana M., de la Torre R. (2021). Contribution of Biotransformations Carried Out by the Microbiota, Drug-Metabolizing Enzymes, and Transport Proteins to the Biological Activities of Phytochemicals Found in the Diet. Adv. Nutr..

[113] Kan J., Wu F., Wang F., Zheng J., Cheng J., Li Y., Yang Y., Du J. (2022). Phytonutrients: Sources, bioavailability, interaction with gut microbiota, and their impacts on human health. Front. Nutr..

[114] Chen T., Dai Y., Hu C., Lin Z., Wang S., Yang J., Zeng L., Li S., Li W. (2024). Cellular and molecular mechanisms of the blood–brain barrier dysfunction in neurodegenerative diseases. Fluids Barriers CNS.

[115] Isabel U.-V., de la Riera M., Belén A., Dolores R.S., Elena G.-B. (2024). A new frontier in neuropharmacology: Recent progress in natural products research for blood–brain barrier crossing. Curr. Res. Biotechnol..

[116] Grabrucker A.M., Ruozi B., Belletti D., Pederzoli F., Forni F., Vandelli M.A., Tosi G. (2016). Nanoparticle transport across the blood brain barrier. Tissue Barriers.

[117] Pardridge W.M. (2005). The blood-brain barrier and neurotherapeutics. NeuroRx.

[118] Kılıç K.D., Garipoğlu G., Çakar B., Uyanıkgil Y., Erbaş O. (2025). Antioxidant-Effective Quercetin Through Modulation of Brain Interleukin-13 Mitigates Autistic-Like Behaviors in the Propionic Acid-Induced Autism Model in Rats. J. Neuroimmune Pharmacol..

[119] Gyawali A., Krol S., Kang Y.S. (2019). Involvement of a Novel Organic Cation Transporter in Paeonol Transport Across the Blood-Brain Barrier. Biomol. Ther..

[120] Carabotti M., Scirocco A., Maselli M.A., Severi C. (2015). The gut-brain axis: Interactions between enteric microbiota, central and enteric nervous systems. Ann. Gastroenterol..

[121] Kandpal M., Indari O., Baral B., Jakhmola S., Tiwari D., Bhandari V., Pandey R.K., Bala K., Sonawane A., Jha H.C. (2022). Dysbiosis of Gut Microbiota from the Perspective of the Gut-Brain Axis: Role in the Provocation of Neurological Disorders. Metabolites.

[122] Plamada D., Vodnar D.C. (2021). Polyphenols-Gut Microbiota Interrelationship: A Transition to a New Generation of Prebiotics. Nutrients.

[123] Enayati A., Soghi A., Butler A.E., Rizzo M., Sahebkar A. (2023). The Effect of Curcumin on the Gut-Brain Axis: Therapeutic Implications. J. Neurogastroenterol. Motil..

[124] Abdolmaleky H.M., Zhou J.R. (2023). Underlying Mechanisms of Brain Aging and Neurodegenerative Diseases as Potential Targets for Preventive or Therapeutic Strategies Using Phytochemicals. Nutrients.

[125] Huang M., Jiang X., Liang Y., Liu Q., Chen S., Guo Y. (2017). Berberine improves cognitive impairment by promoting autophagic clearance and inhibiting production of β-amyloid in APP/tau/PS1 mouse model of Alzheimer’s disease. Exp. Gerontol..

[126] Shi J., Chen J., Xie X., Li Y., Ye W., Yao J., Zhang X., Zhang T., Gao J. (2023). Baicalein-corrected gut microbiota may underlie the amelioration of memory and cognitive deficits in APP/PS1 mice. Front. Pharmacol..

[127] Mancuso R., del Valle J., Modol L., Martinez A., Granado-Serrano A.B., Ramirez-Núñez O., Pallás M., Portero-Otin M., Osta R., Navarro X. (2014). Resveratrol Improves Motoneuron Function and Extends Survival in SOD1G93A ALS Mice. Neurotherapeutics.

[128] Peng K., Tao Y., Zhang J., Wang J., Ye F., Dan G., Zhao Y., Cai Y., Zhao J., Wu Q. (2016). Resveratrol Regulates Mitochondrial Biogenesis and Fission/Fusion to Attenuate Rotenone-Induced Neurotoxicity. Oxid. Med. Cell. Longev..

[129] Sandhir R., Mehrotra A. (2013). Quercetin supplementation is effective in improving mitochondrial dysfunctions induced by 3-nitropropionic acid: Implications in Huntington’s disease. Biochim. Biophys. Acta-Mol. Basis Dis..

[130] Li J., Han Y., Li M., Nie C. (2019). Curcumin Promotes Proliferation of Adult Neural Stem Cells and the Birth of Neurons in Alzheimer’s Disease Mice via Notch Signaling Pathway. Cell. Reprogramm..

[131] Thomas P., Wang Y.-J., Zhong J.-H., Kosaraju S., O’Callaghan N.J., Zhou X.-F., Fenech M. (2009). Grape seed polyphenols and curcumin reduce genomic instability events in a transgenic mouse model for Alzheimer’s disease. Mutat. Res.-Fundam. Mol. Mech. Mutagen..

[132] Cilia R., Laguna J., Cassani E., Cereda E., Pozzi N.G., Isaias I.U., Contin M., Barichella M., Pezzoli G. (2017). Mucuna pruriens in Parkinson disease: A double-blind, randomized, controlled, crossover study. Neurology.

[133] Das S.S., Gopal P.M., Thomas J.V., Mohan M.C., Thomas S.C., Maliakel B.P., Krishnakumar I.M., Sasidharan B.C.P. (2023). Influence of CurQfen^®^-curcumin on cognitive impairment: A randomized, double-blinded, placebo-controlled, 3-arm, 3-sequence comparative study. Front. Dement..

[134] Millar C.L., Iloputaife I., Baldyga K., Norling A.M., Boulougoura A., Vichos T., Tchkonia T., Deisinger A., Pirtskhalava T., Kirkland J.L. (2025). A pilot study of senolytics to improve cognition and mobility in older adults at risk for Alzheimer’s disease. eBioMedicine.

[135] Viña J., Escudero J., Baquero M., Cebrián M., Carbonell-Asíns J.A., Muñoz J.E., Satorres E., Meléndez J.C., Ferrer-Rebolleda J., Cózar-Santiago M.D.P. (2022). Genistein effect on cognition in prodromal Alzheimer’s disease patients. The GENIAL clinical trial. Alzheimers Res. Ther..

[136] Köbe T., Witte A.V., Schnelle A., Tesky V.A., Pantel J., Schuchardt J.-P., Hahn A., Bohlken J., Grittner U., Flöel A. (2017). Impact of resveratrol on glucose control, hippocampal structure and connectivity, and memory performance in patients with mild cognitive impairment. Front. Neurosci..

[137] Moussa C., Hebron M., Huang X., Ahn J., Rissman R.A., Aisen P.S., Turner R.S. (2017). Resveratrol regulates neuro-inflammation and induces adaptive immunity in Alzheimer’s disease. J. Neuroinflamm..

[138] Zhang H., Cao Y., Pei H., Wang H., Ma L., Wang Z., Diao X., Yang Y., Liu N., Wei Y. (2020). Shenmayizhi formula combined with ginkgo extract tablets for the treatment of vascular dementia: A randomized, double-blind, controlled trial. Evid. Based Complement. Alternat. Med..

[139] Santa K., Kumazawa Y., Watanabe K., Nagaoka I. (2024). The potential use of vitamin D3 and phytochemicals for their anti-ageing effects. Int. J. Mol. Sci..

[140] Fakhri S., Piri S., Moradi S.Z., Khan H. (2022). Phytochemicals targeting oxidative stress, interconnected neuroinflammatory, and neuroapoptotic pathways following radiation. Curr. Neuropharmacol..

[141] Yang J., Zhao H., Qu S. (2024). Phytochemicals targeting mitophagy: Therapeutic opportunities and prospects for treating Alzheimer’s disease. Biomed. Pharmacother..

[142] Plano L.M.D., Calabrese G., Rizzo M.G., Oddo S., Caccamo A. (2023). The role of the transcription factor Nrf2 in Alzheimer’s disease: Therapeutic opportunities. Biomolecules.

[143] Heurtaux T., Bouvier D.S., Benani A., Romero S.H., Frauenknecht K.B., Mittelbronn M., Sinkkonen L. (2022). Normal and pathological NRF2 signalling in the central nervous system. Antioxidants.

[144] Ngo V., Duennwald M.L. (2022). Nrf2 and oxidative stress: A general overview of mechanisms and implications in human disease. Antioxidants.

[145] Suzen S., Tucci P., Profumo E., Buttari B., Saso L. (2022). A pivotal role of Nrf2 in neurodegenerative disorders: A new way for therapeutic strategies. Pharmaceuticals.

[146] He W.-J., Lv C.-H., Chen Z., Shi M., Zeng C.-X., Hou D.-X., Qin S. (2023). The regulatory effect of phytochemicals on chronic diseases by targeting Nrf2-ARE signaling pathway. Antioxidants.

[147] Farkhondeh T., Folgado S.L., Pourbagher-Shahri A.M., Ashrafizadeh M., Samarghandian S. (2020). The therapeutic effect of resveratrol: Focusing on the Nrf2 signaling pathway. Biomed. Pharmacother..

[148] Hui Y., Chengyong T., Cheng L., Haixia H., Yuanda Z., Weihua Y. (2018). Resveratrol Attenuates the Cytotoxicity Induced by Amyloid-β(1-42) in PC12 Cells by Upregulating Heme Oxygenase-1 via the PI3K/Akt/Nrf2 Pathway. Neurochem. Res..

[149] Anilkumar S., Wright-Jin E. (2024). NF-κB as an inducible regulator of inflammation in the central nervous system. Cells.

[150] Sivamaruthi B.S., Raghani N., Chorawala M., Bhattacharya S., Prajapati B.G., Elossaily G.M., Chaiyasut C. (2023). NF-κB pathway and its inhibitors: A promising frontier in the management of Alzheimer’s disease. Biomedicines.

[151] Chauhan A., Islam A.U., Prakash H., Singh S. (2022). Phytochemicals targeting NF-κB signaling: Potential anti-cancer interventions. J. Pharm. Anal..

[152] Olivera A., Moore T.W., Hu F., Brown A.P., Sun A., Liotta D.C., Snyder J.P., Yoon Y., Shim H., Marcus A.I. (2012). Inhibition of the NF-κB signaling pathway by the curcumin analog, 3,5-Bis(2-pyridinylmethylidene)-4-piperidone (EF31): Anti-inflammatory and anti-cancer properties. Int. Immunopharmacol..

[153] Zhang J., Zheng Y., Luo Y., Du Y., Zhang X., Fu J. (2019). Curcumin inhibits LPS-induced neuroinflammation by promoting microglial M2 polarization via TREM2/TLR4/NF-κB pathways in BV2 cells. Mol. Immunol..

[154] Su Z., Guo Y., Huang X., Feng B., Tang L., Zheng G., Zhu Y. (2021). Phytochemicals: Targeting mitophagy to treat metabolic disorders. Front. Cell Dev. Biol..

[155] Zeng K., Yu X., Mahaman Y.A.R., Wang J.-Z., Liu R., Li Y., Wang X. (2022). Defective mitophagy and the etiopathogenesis of Alzheimer’s disease. Transl. Neurodegener..

[156] Liang J.H., Yu H., Xia C.P., Zheng Y.H., Zhang Z., Chen Y., Raza M.A., Wu L., Yan H. (2024). Ginkgolide B effectively mitigates neuropathic pain by suppressing the activation of the NLRP3 inflammasome through the induction of mitophagy in rats. Biomed. Pharmacother..

[157] Chen F. (2024). Inhibiting Pink1/Parkin-mediated mitophagy enhances the anticancer effects of quercetin in hepatocellular carcinomaf. Biochem. Biophys. Res. Commun..

[158] Kumar A., Nirmal P., Kumar M., Jose A., Tomer V., Oz E., Proestos C., Zeng M., Elobeid T., Sneha K. (2023). Major phytochemicals: Recent advances in health benefits and extraction method. Molecules.

[159] Davinelli S., Maes M., Corbi G., Zarrelli A., Willcox D.C., Scapagnini G. (2016). Dietary phytochemicals and neuro-inflammaging: From mechanistic insights to translational challenges. Immun. Ageing.

[160] Chihomvu P., Ganesan A., Gibbons S., Woollard K., Hayes M.A. (2024). Phytochemicals in drug discovery—A confluence of tradition and innovation. Int. J. Mol. Sci..

[161] Paul J.K., Azmal M., Haque A.S.N.B., Talukder O.F., Meem M., Ghosh A. (2024). Phytochemical-mediated modulation of signaling pathways: A promising avenue for drug discovery. Adv. Redox Res..

[162] Wu W., Huang J., Han P., Zhang J., Wang Y., Jin F., Zhou Y. (2023). Research Progress on Natural Plant Molecules in Regulating the Blood–Brain Barrier in Alzheimer’s Disease. Molecules.

[163] Ahlawat J., Barroso G.G., Asil S.M., Alvarado M., Armendariz I., Bernal J., Carabaza X., Chavez S., Cruz P., Escalante V. (2020). Nanocarriers as Potential Drug Delivery Candidates for Overcoming the Blood–Brain Barrier: Challenges and Possibilities. ACS Omega.

